# Differential impact of classical and non-canonical NF-κB pathway-related gene expression on the survival of breast cancer patients

**DOI:** 10.7150/jca.34302

**Published:** 2019-08-28

**Authors:** Nancy Adriana Espinoza-Sánchez, Balázs Győrffy, Ezequiel M. Fuentes-Pananá, Martin Götte

**Affiliations:** 1 Unidad de Investigación en Virología y Cáncer, Hospital Infantil de México Federico Gómez, C.P. 06720, Ciudad de México, México; 2 MTA TTK Lendület Cancer Biomarker Research Group, Institute of Enzymology, Hungarian Academy of Sciences, and Semmelweis University 2nd Dept. of Pediatrics, Budapest, Hungary.; 3 Department of Gynecology and Obstetrics, Münster University Hospital, Münster, Germany.

**Keywords:** Breast cancer, NF-κB, inflammation, survival analysis, prognosis, KM plotter.

## Abstract

Inflammation is a well-known driver of carcinogenesis and cancer progression, often attributed to the tumor microenvironment. However, tumor cells themselves are capable of secreting a variety of inflammatory molecules, leading to the activation of specific signaling pathways that promote tumor progression. The NF-κB signaling pathway is one of the most important connections between inflammation and tumorigenesis. NF-κB is a superfamily of transcription factors that plays an important role in several types of hematological and solid tumors, including breast cancer. However, the role of the NF-κB pathway in the survival of breast cancer patients is poorly studied. In this study, we analyzed and related the expression of both canonical and alternative NF-κB pathways and selected target genes with the relapse-free and overall survival of breast cancer patients. We used the public database Kaplan-Meier plotter (KMplot) which includes gene expression data and survival information of 3951 breast cancer patients. We found that the expression of *IKKα* was associated with poor relapse-free survival in patients with ER-positive tumors. Moreover, the expression of* IL-8* and *MMP-1* was associated with poor relapse-free and overall survival. In contrast, expression of *IKKβ*, *p50,* and *p65* from the canonical pathway, and *NIK* and *RELB* from the alternative pathway correlated with better relapse-free survival also when the patients were classified by their hormonal and nodal status. Our study suggests that the expression of genes of the canonical and alternative NF-κB pathways is ultimately critical for tumor persistence. Understanding the communication between both pathways would help to find better therapeutic and prophylactic targets to prevent breast cancer progression and relapse.

## Introduction

In recent years, the clinical problem of breast cancer diagnosis and treatment gained increasing public attention. Breast cancer is the most common cause of cancer mortality in young productive women, especially in developing countries 1. The current problem with breast cancer is that it is a highly heterogeneous disease both inter- and intra-tumoral and it is classified into different subtypes with variable clinical outcomes 2, 3. The markers and clinical parameters known to date (tumor size, expression of estrogen and progesterone receptors, and HER-2/neu, histological or nuclear grade) are not applicable in all clinical stages and between the patients, and limit their utility to decide the most appropriate chemotherapeutic treatment since they have shown a weak association with the clinical response of patients 4. The involvement of axillary lymph nodes has been shown to be the most important predictor of disease-free survival and overall survival in breast cancer 5, 6. Approximately 30% of lymph node-negative patients have a relapse within 10 years, compared with around 70% of patients with axillary nodal positive involvement 7, 8. We need to understand more about this disease in order to find specific biomarkers and potential drug targets that help to change the prognostic of the patient.

In addition, breast cancer cells are surrounded by a microenvironment, the tumor microenvironment (TME), composed of cells of the immune system and connective tissue. It has been proposed that the communication between the neoplastic cell and their TME through soluble secreted factors or cell-cell contacts promotes tumor progression by interference with the function of cells of the immune system 9, 10. Even though a patient may be initially diagnosed with a tumor type indicative of a good prognosis, the influence of the microenvironment and the tumor crosstalk could change the course of the disease. Nowadays, the communication between cancer and immune cells has and is being extensively studied, with novel therapeutic drugs developed against immune checkpoint controls 11, 12. Currently, the FDA has approved the use of Nivolumab and Ipilimumab, targeting PD-1 and CTLA-4 respectively, to treat melanoma 13. The link between chronic inflammation and carcinogenesis was already proposed by Rudolf Virchow as early as 1863 14 and in spite of the recent advances, little is known about the signaling pathways that link these processes and its participation in the prognosis of patients with breast cancer. For example, in inflammatory breast cancer (IBC), an aggressive form of breast cancer, overexpression of Her2 and NF-κB (nuclear factor kappa-light-chain-enhancer of activated B cells) is associated with a poor prognosis 15.

NF-κB refers to a family of five different DNA-binding proteins which are key regulators of innate and adaptive immune responses. Furthermore, NF-κB is also involved in the regulation of multiple pathogenetically relevant processes in epithelial cells such as proliferation, angiogenesis, migration, invasion, and metastasis (all processes considered as hallmarks of cancer) through the activation of cytokines like IL-8 or metalloproteases (MMPs) 16, 17. These transcription factors are NF-κB1 (p50/p105), NFκB2 (p52/p100), RelA (p65), RelB, and c-Rel; which can homo or hetero-dimerize to allow DNA binding and activate transcription 17. NF-κB can be activated by multiple ligands, including cytokine receptors, PAMPs (Pathogen-Associated Molecular Patterns), physiological stress, TNF (Tumor Necrosis Factor), and members of the TNF receptor family such as CD40, and BAFF (B cell-activating factor of the TNF family). There are two pathways by which the signal is transduced: 1) the classical (also named canonical), and 2) the alternative or non-canonical pathway. Both pathways have in common that receptor activation induces the phosphorylation and subsequent degradation of IκB proteins (the NF-κB inhibitors), however, while the classical pathway mediates this effect through the IKK complex, the alternative pathway acts through NIK (NF-κB-inducing kinase) and IKKα 17-19. Upon IκB degradation, NF-κB translocates to the nucleus to act as a transcription factor. Also, different NF-κB dimers represent canonical and non-canonical pathways, for instance, p50:p65 are common to classical activation and p52:RelB to the alternative pathway 17-19.

An active NF-κB pathway is decisive to explain the inflammatory profile of IBC, as well as the high metastatic potential of this form of breast cancer, through secretion of cytokines, growth factors, and proteases. An NF-κB transcriptional signature has been observed in IBC but also in ER-positive luminal breast cancers 20. The NF-κB pathway has been associated with early events during oncogenesis 21.* In vitro* data by Espinoza-Sánchez *et al* have suggested that the NF-κB pathway is a key regulator of intra-tumoral communication responsible for tumor cell plasticity in breast cancer 22. In a cohort of 59 patients with primary breast cancer, levels of phosphorylated p65 correlated with HER2 expression, tumor size, grade and presence of metastases 23, 24. In a tissue microarray of 376 patients with invasive ductal breast cancer, Bennett *et al* demonstrated that the activation of the classical NF-κB pathway correlates with poor outcome 25. However, so far, a comprehensive analysis of the impact of the classical and non-canonical NF-κB pathways is missing.

In this study, to assess the relevance of the activity of key members and targets of the NF-κB pathway for relapse-free and overall survival of breast cancer patients, we performed Kaplan-Meier plot survival analysis using the online database www.kmplot.com/breastcancer 26. This web-tool allows for the selection of patients, which can be filtered by receptors status, lymph node involvement, molecular classification, and others. We have found that the expression of members of the canonical and alternative NF-κB pathway influence prognosis in breast cancer patients independent from their classification (molecular, grade or LN status). Interestingly, IKKα, a common component of the canonical and alternative pathways emerges as a possible candidate to determinate the outcome of the patient as well as the downstream regulatory targets IL-8 and MMP-1 and may represent potential therapeutic targets, especially for IKKα in ER-positive tumors.

## Material and methods

### Kaplan-Meier plots

In order to analyze the prognostic value of a member of the NF-κB pathway in the relapse-free survival of patients with breast cancer, we used the publically available gene expression database Kaplan Meier plotter 27. This database was established using gene expression data and survival information downloaded from Gene Expression Omnibus (GEO) 27 and contains patient samples from Memorial Sloan Kettering Cancer Institute, New York, USA; the Breast International Group (BIG) 1-98 that includes patients of different European and Latin American countries, and the USA; Radboud University Nijmegen Medical Centre, Nijmegen, The Netherlands; Technical University Munich, Germany; National Cancer Institute, Bari, Italy; Institute of Oncology, Ljubljana, Slovenia; Department of Obstetrics, Gynecology of the Johannes Gutenberg University Mainz, Germany; John Radcliffe Hospital, Oxford, United Kingdom; Guys Hospital, London, United Kingdom; Uppsala University Hospital, Uppsala, Sweden; Institut Gustave Roussy, Villejuif, France; Karolinska Institute, Stockholm, Sweden; Centre Rene´ Huguenin, Saint-Cloud, France; Netherlands Cancer Institute, Netherlands; Stanford University, USA; National University Hospital, Singapore; and Erasmus Medical Center, Rotterdam, The Netherlands 27-42. Currently, this program has relapse-free survival data of 3,955 patients and overall survival data of 1,402 patients downloaded from GEO and EGA (European Genome-Phenome Archive). The package "survival" was used in the R programming environment to plot Kaplan-Meier survival curves and the number-at-risk 27. The 'median' cutoff was used to split patient groups into high and low expression cohorts and these were compared using Cox regression analysis. Patients were stratified by ER status, HER2 status (n=1,872), lymph node status, molecular classification, Pietenpol subtype, and grading. We studied some components of the NF-κB pathway and visualized the correlation to survival by drawing Kaplan-Meier survival plots. The Affymetrix IDs utilized for the genes were: 209666_s_at-IKKα, 209341_s_at-IKBKB, 209239_at-p50/NFKB1, 201783_s_at-p65/RELA, 205192_at-NIK, 207535_s_at-p52, 205205_at-RELB, 202859_x_at-IL-8, 205207_at-IL6, and 204475_at-MMP-1. Finally, the False Discovery Rate (FDR) was computed to correct for multiple hypothesis testing. The percentage of patients tabulated in table 1 is a result from 37 different datasets, 35 from GEO (GSE1456, GSE2034, GSE2990, GSE3494, GSE4922, GSE6532, GSE7390, GSE11121, GSE12093, GSE5327, GSE9195, GSE16391, GSE12276, GSE2603, GSE17705, GSE21653, GSE16446, GSE17907, GSE19615, GSE20685, GSE20711, GSE26971, GSE31448, GSE31519, GSE20194, GSE20271, GSE32646, GSE18728, GSE23988, GSE41998, GSE16716, GSE42568, GSE45255, GSE37946, GSE4611) and 2 from EGA (E-MTAB-365, E-TABM-43).

### Cell culture

All human breast cancer cell lines except SUM149 were purchased from ATCC/LGC Promochem (Wesel, Germany). T47D, MDA-MB-453, MDA-MB-468, MDA-MB-231, and SKBR3 cells were maintained in DMEM containing 10% FCS, 1% glutamine and 1% penicillin/streptomycin in a humidified atmosphere of 7.5% CO_2_ at 37°C. MCF-7 cells were maintained in RPMI containing 10% FCS, 1% glutamine and 1% penicillin/streptomycin in a humidified atmosphere of 5% CO_2_ at 37°C. BT474 cells were maintained in RPMI containing 20% FCS, 1% glutamine and 1% penicillin/streptomycin and 0.01 mg/ml insulin in a humidified atmosphere of 5% CO_2_ at 37°C. The inflammatory breast cancer cell line SUM149 (a kind gift from Dr. Bonnie Sloane, Wayne State University, Detroit, MI, USA) was maintaining in HAM´s containing 10% FCS, 1% glutamine and 1% penicillin/streptomycin in a humidified atmosphere of 7.5% CO_2_ at 37°C. To activate NF-κB, BrC cells were plated at a density of 20,000 cells in 8 well chamber slides (Nunc, Wiesbaden, Germany) to perform immunofluorescence microscopy (IF). Once cells attached to the plate, supernatants were discarded, cells were rinsed with PBS, and then stimulated with 40 ng/mL of human recombinant human TNF-α (R&D Systems, Wiesbaden, Germany.) in culture medium for 40 min., followed by processing for immunofluorescence microscopy as indicated below.

### Quantitative real-time PCR

Total RNA was isolated from the cultured human breast cancer cells MCF-7, T47D, BT474, SKBR3, MDA-MB-453, MDA-MB-468, MDA-MB-231, and SUM149 using the innuPREP RNAMini Kit (Biometra, Göttingen, Germany) according to the manufacturer's instructions and RNA was reverse transcribed into cDNA using the First Strand cDNA Synthesis Kit (Thermo Fisher, Schwerte, Germany), random hexamer primers and M-MuLV reverse transcriptase. Quantitative real-time PCR was conducted in triplicates for each gene of interest using SYBR Green dye (Thermo Fisher) and gene expression levels were measured in an ABI PRISM 7300 SequenceDetection System (Thermo Fisher). For transcriptional analysis, the RT-PCR products were quantified by the fluorescent method using the 2-ΔCt value. To normalize gene expression, Cycle threshold (Ct) values from each sample were normalized with its corresponding *β-ACTIN* Ct. Normalized values were converted to log base 2 before data was evaluated by statistical analysis. Melting curve analysis was performed to confirm specific product amplification. Primer sequences were confirmed by NCBI BLAST analysis and are listed in the supplementary file Table S8.

### Confocal immunofluorescence microscopy

TNF-α-stimulated or unstimulated cell lines grown on chamber slides were fixed with 3.7% PBS-buffered formaldehyde and permeabilized with PBS/0.1% Triton-X100. Nonspecific binding was blocked with PBS/1% Aurion BSA-c (DAKO, Glostrup, Denmark). Coverslips were subsequently incubated for 1 hr with rabbit anti-NF-κB p65 (C22B4) mAb (Cell Signaling Technology, Frankfurt a.M., Germany) diluted 1:1,000 in PBS/1% Aurion BSA, followed by 3 x 5 min washes with PBS. Subsequently, the samples were incubated with Alexa Fluor 488 conjugated donkey anti-rabbit antibody (Invitrogen, Karlsruhe, Germany) diluted 1:500, in PBS/1% Aurion BSA-c. for 30 min. Samples were washed 2x for 5 min in PBS, stained with 300 nM DAPI (Sigma, Deisenhofen, Germany) for 1 min, followed by a brief wash with PBS and slide mounting, Slides were analyzed with a confocal microscope (Axioskop; Carl Zeiss, Göttingen, Germany) with × 40 magnification and a 0.75 numerical aperture oil immersion objective.

### Protein interaction network analysis

STRING v11 (http://string-db.org/) was used to generate in silico protein interaction networks for the gene products that we analyzed in KMplot, IKKα (CHUK), IKBKB, p50/NFKB1, p65/RELA, NIK (MAP4K4), p52 (NFKB2), RELB, IL-8 (CXCL8), IL6, and MMP-1. All interactions are predicted with a high confidence threshold of 0.700, and all active predictive methods were allowed. For the enrichment analysis, STRING implements well-known classification systems such as Gene Ontology (GO) and KEGG (Kyoto Encyclopedia of Genes and Genomes) 43.

### Statistical analysis

For survival analysis, in the R statistical environment, we utilized the Kaplan-Meier-Plotter database via the statistical package "survival" to calculate Kaplan-Meier survival curves and the number-at-risk. Furthermore, the hazard ratio (and 95% confidence intervals) and log-rank P were calculated for each gene 27. The FDR was computed using the brainwaver library in R. For analysis of qPCR results, the Prism software version 5.01 (GraphPad) was used for statistical analysis. A oneway analysis of variance (ANOVA) test with the Tukey as a post hoc test was applied to more than two groups of data, and the non-parametric Kruskal-Wallis test with Dunnett's as post hoc test was applied to more than two groups in case the data lack normality and/or homogeneity of variance. Significant P-values are indicated as follows: ≤0.05 by one asterisk *, ≤0.01 by two asterisks ** and ≤ 0.001 by three asterisks ***.

## Results

### Expression of IKKα is associated with poor relapse-free survival, while representative genes from the alternative pathway are associated with better relapse-free and overall survival in breast cancer patients

We analyzed the correlation between the expression of common and unique members of both canonical and alternative NF-κB pathways and some of their transcriptional targets with the relapse-free survival (RFS) and overall survival (OS) of breast cancer patients. A total of 3,951 for RFS and 1,402 for OS patients without any classification were analyzed using the online database www.kmplot.com/breastcancer, **Table 1** displays the clinicopathological characteristics of the investigated patients.

**Figure 1:**
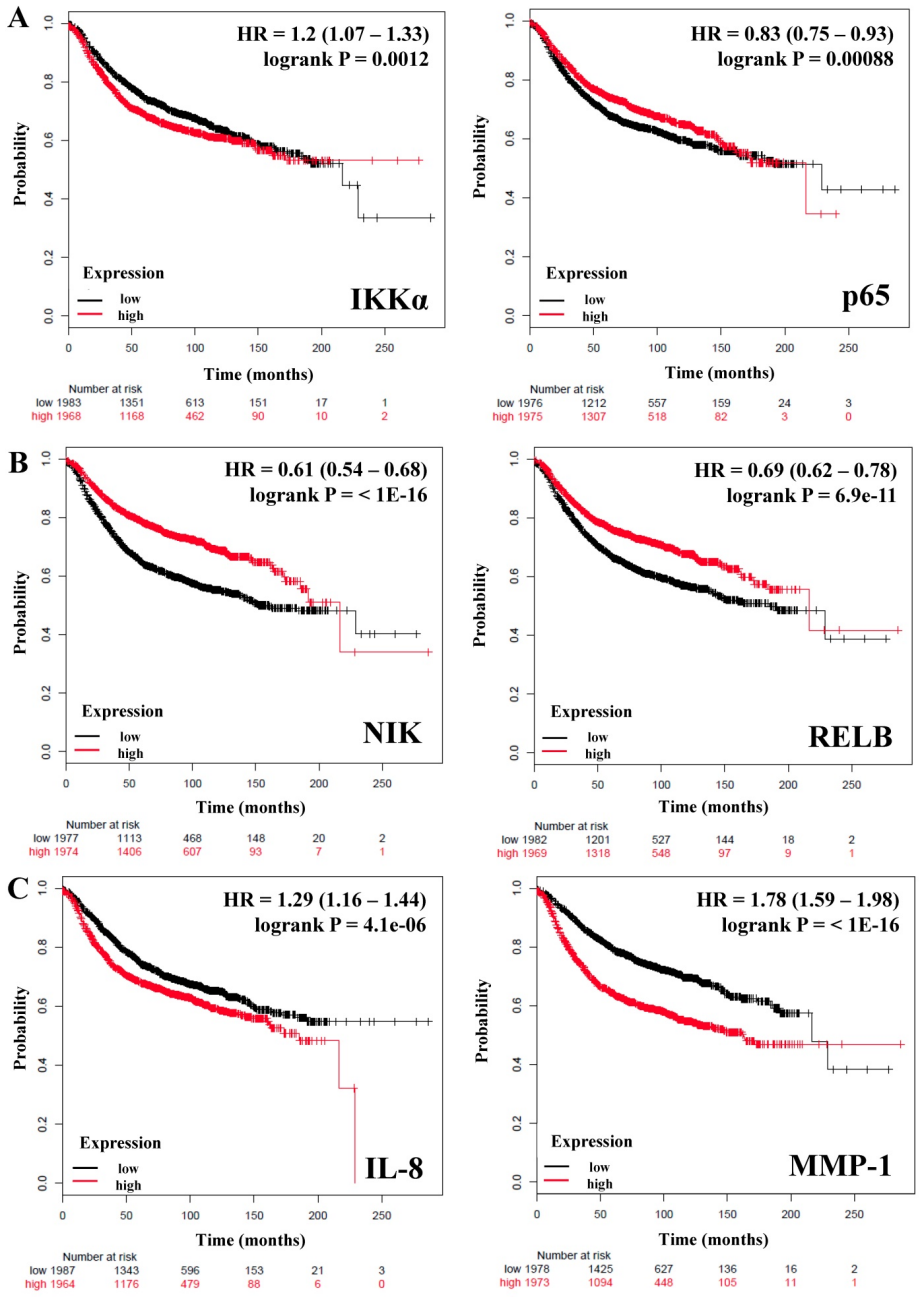
** The prognostic value of the expression of genes associated with the NF-κB pathway.** Kaplan-Meier relapse-free survival curves are plotted for all breast cancer patients (n=3951). A) IKKα, a common component of the canonical and alternative pathways and p65 from the classical NF-κB pathway. B) Genes of the alternative NF-κB pathway. C) Targets genes of the NF-κB pathway. Log-rank p values and hazard ratios (HRs; 95 % confidence interval in parentheses) are shown. The desired Affymetrix IDs are: 209666_s_at-IKKα, 201783_s_at-p65/RELA, 205192_at-NIK, 205205_at-RELB, 202859_x_at-IL-8, and 204475_at-MMP-1.

**Table 2** shows the HR (Hazard ratio) and *P* values of 10 genes: *IKKβ, p50* and *p65* from the canonical pathway; *NIK, p52,* and *RELB* from the alternative pathway; *IKKα* which is common to both canonical and non-canonical pathways, and *IL-8, IL-6, MMP-1* as downstream targets controlled by these pathways. We observed that only *IKKα* correlates with poor RFS in breast cancer patients (HR = 1.2; *P* = 0.0012) (**Figure 1A**), we did not find a correlation of this gene with the OS (**Table 2**). In contrast *IKKβ* and *p50* from the canonical pathway and the expression of *NIK* and *RELB* from the alternative pathway correlated with better RFS and OS in all patients (HR = <1, *P* = <0.05) (**Table 2 and Figure 1B**). The expression of *p65* HR = 0.83; *P* = 0.00088 (**Figure 1A**) and p52 HR = 0.8; *P* = 5.7e-05 (**Table 2**) correlated also with better RFS, but not with OS. Since inflammatory factors, chemokines and some proteases are important targets of the NF-κB pathway 17, 44, 45, we analyzed the expression of *IL-8, IL-6,* and *MMP-1* (also factors associated with the breast cancer tumor microenvironment and progression 46) and correlated them with the RFS and OS of the patients. *IL-8* and *MMP-1* were associated with poor RFS and OS, *IL-8* with a HR = 1.29 (*P* = 4.1e-06) regarding RFS and HR = 1.52 (*P* = 0.00013) regarding OS; and *MMP-1* with a HR = 1.78 (*P* = <1E-16) regarding RFS and HR = 1.6 (*P* = 1.6e-05) regarding OS (**Figure 1C and Table 2**). *IL-6* (HR* =* 0.81,* P* = 0.00021) was associated with a better RFS but, not with OS (**Table 2**). Altogether, these data support that expression of genes related to both the canonical and alternative pathways of NF-κB influence the prognosis of breast cancer patients.

### Members of the canonical and alternative NF-κB pathway are associated with better relapse-free survival of breast cancer patients independent of estrogen receptor status

**Figure 2:**
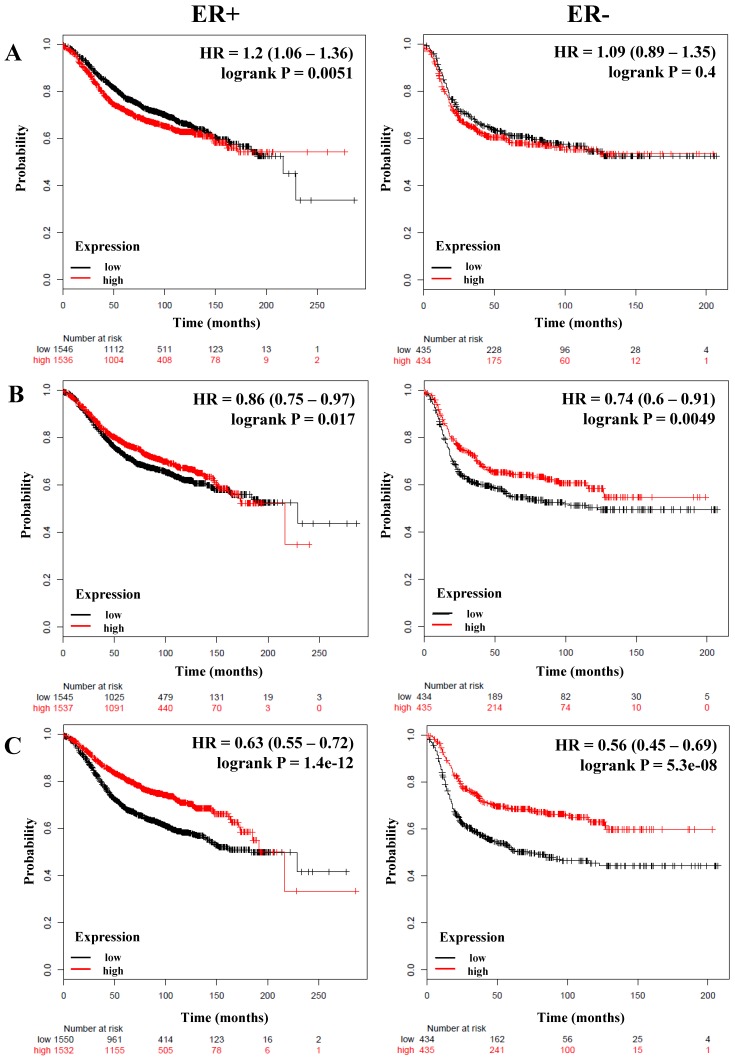

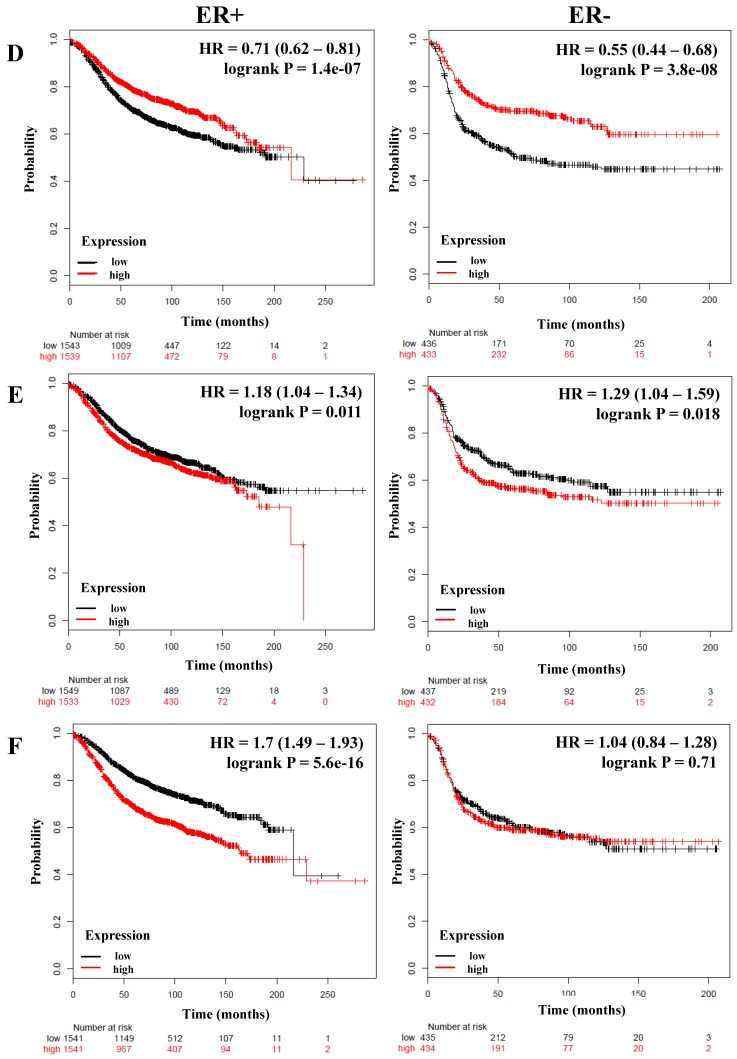
** The prognostic value of the expression of genes associated with the NF-κB pathway based on ER (estrogen receptor) status.** Kaplan-Meier relapse-free survival curves are plotted for breast cancer patients with ER+ (n=3082) and ER- (n=869), and the expression of A) *IKKα*, B) *p65*, C) *NIK*, D) *RELB*, E) *IL-8*, and F) *MMP-1*. Log-rank p values and hazard ratios (HRs; 95 % confidence interval in parentheses) are shown.

We next analyzed if there was a correlation between members of the NF-κB pathway and the RFS and OS of breast cancer patients stratified by estrogen receptor status. A total of 3,082 breast cancer patients with RFS data and 1,044 with OS data and estrogen receptor (ER) positive status; and 869 (RFS) and 358 (OS) patients with ER-negative status were analyzed (**Table 3**). Again we could observe that *IKKα* correlated with poor RFS only in ER-positive patients (HR = 1.2; *P* = 0.0051), but we did not find an association with OS (**Figure 2A** and **Table 3**). In contrast, *p50, p65, p52, NIK, RELB* and, *IL-6* were associated with better RFS in all patients regardless of their ER status (**Figure 2B, C, and D, Table 3**). Also, *p50* and *RELB* correlated with better OS in both ER-positive and ER-negative patients (**Table 3**). The expression of *IKKβ* was associated with better RFS only in ER-positive tumors (HR = 0.74; *P* = 5.9e-06). The expression of *IL-8* was associated with poor RFS and OS in patients with ER-positive tumors (HR = 1.18; *P* = 0.011; HR = 1.36; *P* = 0.018, respectively) and also correlated with poor RFS in ER-negative tumors (HR = 1.29; *P* = 0.018) (**Figure 2E** and** Table 3**). Interestingly, *MMP-1* was associated with poor RFS and OS only in patients with ER-positive tumors (HR = 1.7; *P* = 5.6e-16 and HR = 1.61; *P* = 0.00027, respectively) (**Figure 2F** and **Table 3**).

We also wanted to analyze patients based on the Luminal A molecular classification, which is based on the expression of the estrogen and progesterone receptor and is associated to a better outcome in breast cancer patients 2, 3. A total of 1,933 patients with RFS data and 611 with OS data were analyzed. We could corroborate that even classifying patients in this way, the expression of *IKKα* (HR = 1.2; *P* = 0.039), *IL-8* (HR = 1.2; *P* = 0.035), and MMP-1 (HR = 1.89; *P* = 4.6e-13) correlates with poor RFS; *MMP-1* expression also correlated with poor OS (**Table S1**). Again, *IKKβ*, *p50, p65, p52, NIK, RELB* and, *IL-6* were associated with better RFS; p50 and *RELB* were associated with better OS (**Table S1**). These results suggest that the expression of several members of the canonical or alternative NF-κB pathway has a positive prognostic value in breast cancer that is independent of estrogen receptor status.

### The association between the expression of members of the canonical and alternative NF-κB pathways and good prognosis in breast cancer does not depend on the molecular classification

We next wanted to evaluate if the association of genes of the NF-κB pathway with a good prognosis in breast cancer patients was associated with a particular molecular subtype of the disease.

Since the canonical and alternative pathway have been associated with basal or triple-negative breast cancer (TNBC), which have the worst outcome in patients 47-50, we analyzed all the aforementioned genes in 618 patients with RFS and 241 with OS data classified with the intrinsic basal molecular subtype. Our results shown that only the expression of *p50* from the canonical pathway was associated with better RFS with an HR = 0.65; *P* = 0.00099. In addition, the expression of all the genes associated with the alternative pathway correlated with better RFS: *NIK* HR = 0.54; *P* = 2.4e-06, *p52* HR = 0.69; *P* = 0.0039 and *RELB* HR = 0.54; *P* = 1.8e-06. *NIK* and *RELB* also were associated with better OS. Of the target genes analyzed, *IL-8* was associated with poor RFS with an HR = 1.46; *P* = 0.0035. *MMP-1* expression was associated with poor OS (HR = 1.65; *P* = 0.048), but not with RFS (**Table S2**).

In 2011, Pietenpol and coworkers classified TNBC into 6 subtypes, among them the immunomodulatory subtype which was enriched for gene ontologies of immune cell-related processes 51. Due to the strong relationship between the NF-κB pathway and immune processes we analyzed the relationship between this pathway and RFS and OS of breast cancer patients classified as the immunomodulatory subtype (n= 203 with RFS data and n=100 with OS data). We found that *p50* with a HR = 0.51; *P* = 0.033 and *NIK* HR = 0.51; *P* = 0.033 correlated with better RFS in the patients. Not surprisingly, we observed that IL-8 was associated to poor RFS and OS (HR = 2; *P* = 0.024, HR = 4.11; *P* = 0.007; respectively) and we note that it had the highest HR using this classification. Interestingly, MMP-1 correlated with better RFS (HR = 0.49; *P* = 0.02) (**Table S3**). Furthermore, we analyzed the Pietenpol Basal-like 1 classification in 171 breast cancer patients with RFS data (panel A) and 58 with OS data (panel B). Of all the genes analyzed, only *NIK* has an association with better RFS and OS (**Supplementary Figure S1**), the other genes had no association with RFS or OS (data not shown).

### The expression of members of the canonical and alternative NF-κB pathway correlates with better relapse-free survival of breast cancer patients and is independent of their lymph node status

**Figure 3:**
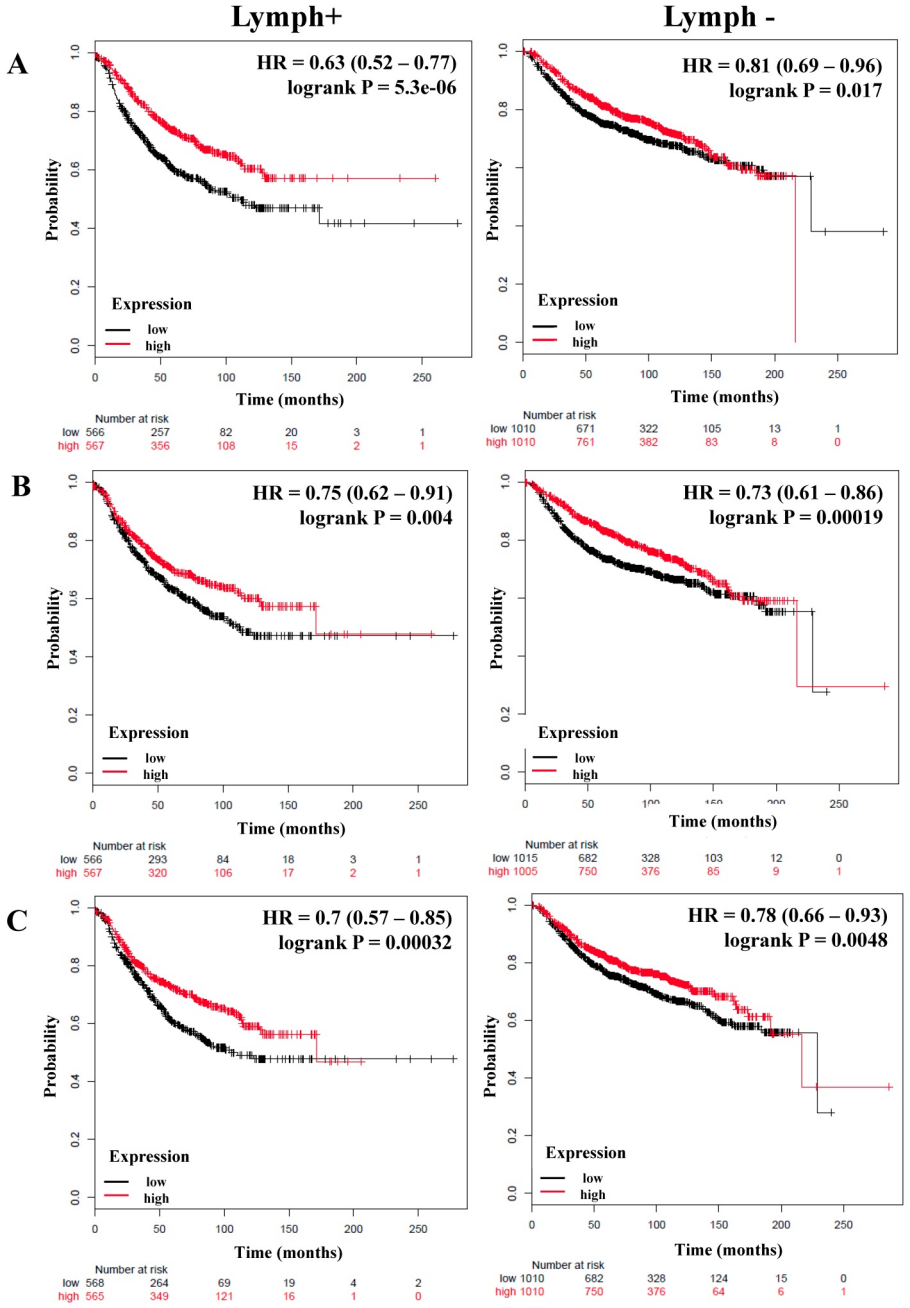

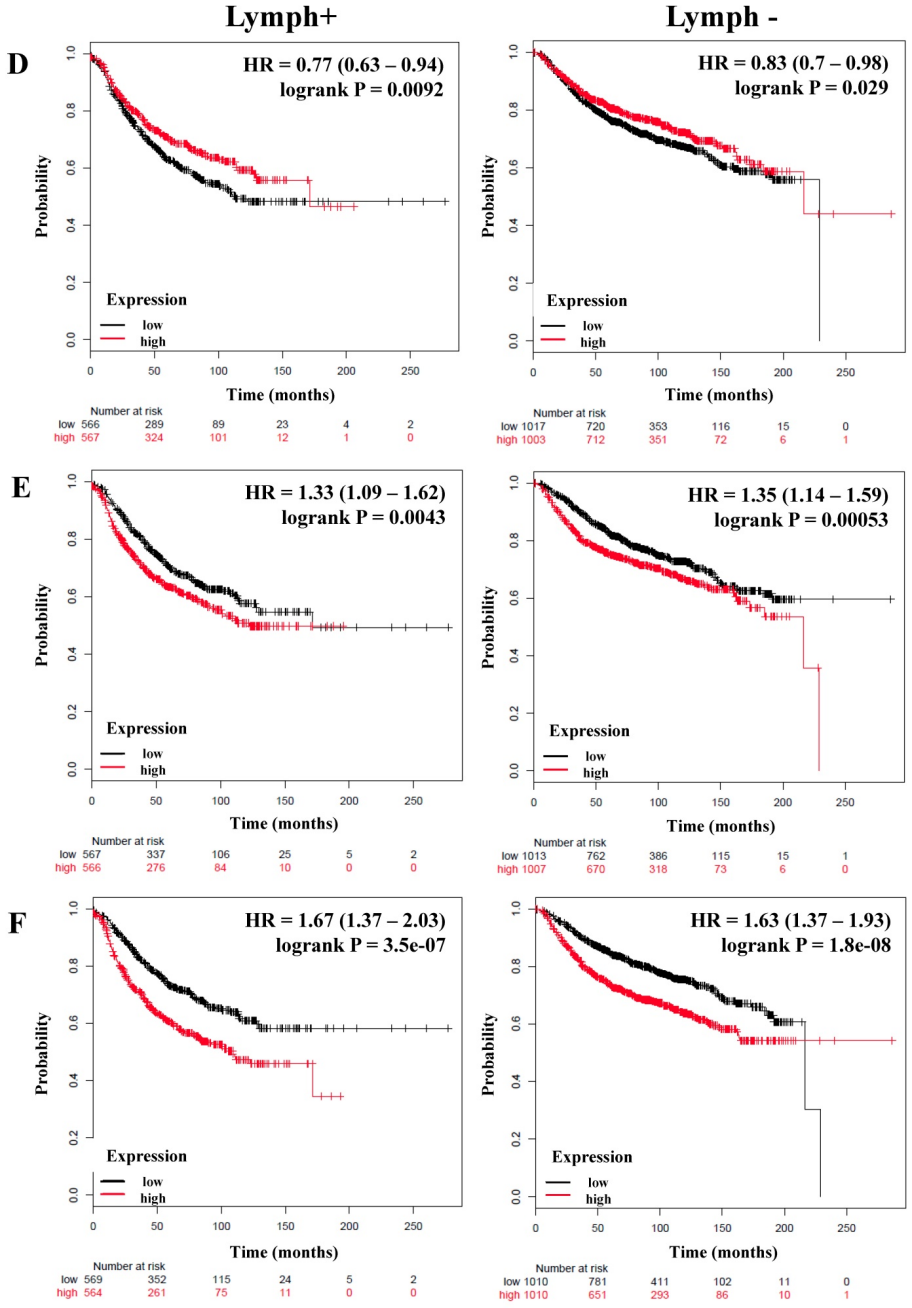
** The prognostic value of the expression of genes associated with the NF-κB pathway based on the Lymph node status.** Kaplan-Meier relapse-free survival curves are plotted for breast cancer patients with Lymph+ (n=1133) and Lymph- (n=2020), and the expression of A) *IKKβ*, B) *p50*, C) *NIK*, D) *RELB*, E) *IL-8*, and F) *MMP-1*. Log-rank p values and hazard ratios (HRs; 95 % confidence interval in parentheses) are shown.

We next determined the correlation of the NF-κB pathway and selected targets with nodal status, analyzing a total of 1,133 breast cancer patients with lymph node (LN) positive and 2020 with lymph node-negative status for RFS (**Table 4**). Although the HR of *IKKα* and *p65* has a high score, its expression was not significantly related to the RFS of patients regardless of nodal status (**Table 4**). However, the expression of *IKKβ* (LN-positive: HR = 0.63, *P* = 5.5e-06; LN-negative: HR = 0.81, *P* = 0.017), *p50* (LN-positive: HR = 0.75, *P* = 0.004; LN-negative: HR = 0.73, *P* = 0.00019), *NIK* (LN-positive: HR = 0.7, *P* = 0.00032; LN-negative: HR = 0.78, *P* = 0.0048), p52 (LN-positive: HR = 0.67, *P* = 6.8e-05; LN-negative: HR = 0.83, *P* = 0.028), and *RELB* (LN-positive: HR = 0.77, *P* = 0.0092; LN-negative: HR = 0.83, *P* = 0.029) correlated with better RFS in all patients independent of their LN status (**Figure 3A-D, Table 4**). In samples for which OS data were available, (LN-positive n = 313 and LN-negative n = 594), *IKKβ* was associated with better OS in LN-positive patients. The same association was seen for p50 and *NIK* in LN-negative patients (**Table 4**).

Interestingly, the expression of important targets of the NF-κB pathway such as *IL-8* and *MMP-1* has a poor RFS in the patients regardless of nodal status (**Figure 3E, F**). Of these genes, only MMP-1 correlated with poor OS in LN-negative tumors (**Table 4**). The grade of a tumor is an important parameter to analyze the treatment and prognosis of a breast cancer patient, frequently associated with LN status 52. Analyzing the patients according to grade 3 status (n = 903), the expression of *p50*, *NIK, p52*, and *RELB* correlated with better RFS (**Table S4**). Analyzing the total of patients with available OS data (n=503), the expression of *NIK* and *RELB* were associated with better OS (**Table S4**).

### The expression of *NIK*,* RELB*, *IL-8*, and *MMP-1* are associated with the basal subtype in human breast cancer cell lines

**Figure 4:**
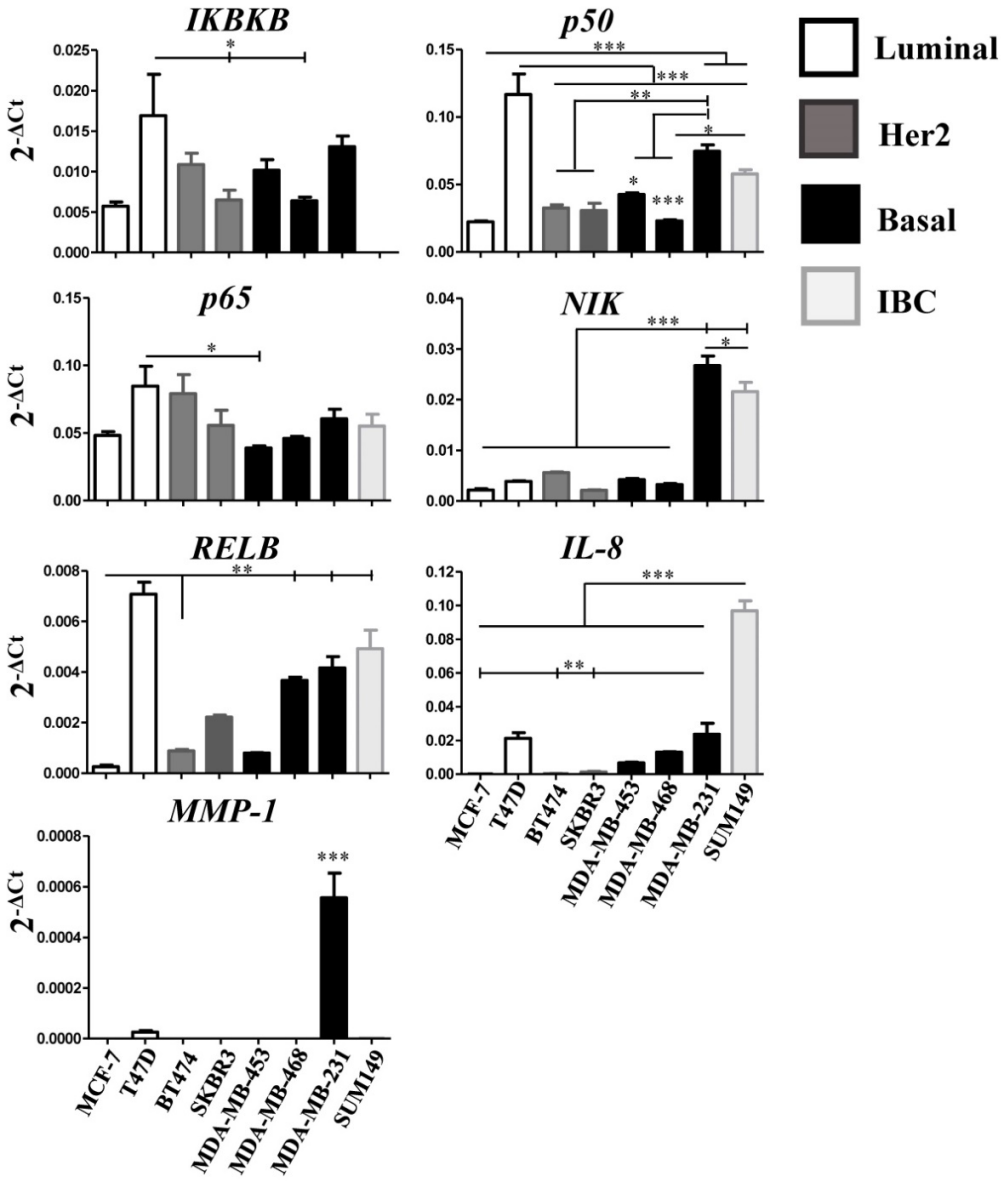
** Relative expression of NF-κB pathway-related genes in breast cancer cell lines.** *IKBKB*, *p50*, *p65*, *NIK*, *RELB*, *IL-8*, and *MMP-1* gene relative expression was quantified by qRT-PCR in 7 breast cancer cell lines representative of the luminal, basal and Her2-positive subtype, and in one inflammatory breast cancer (IBC) cell line. Individual experiments were normalized against *_β_-ACTIN* and relative expression was represented by 2-ΔCt. Data represent the mean ± SEM (standard error of the mean) from one experiment in triplicates; *P<0.05, **P<0.01 and ***P<0.001.

**Figure 5:**
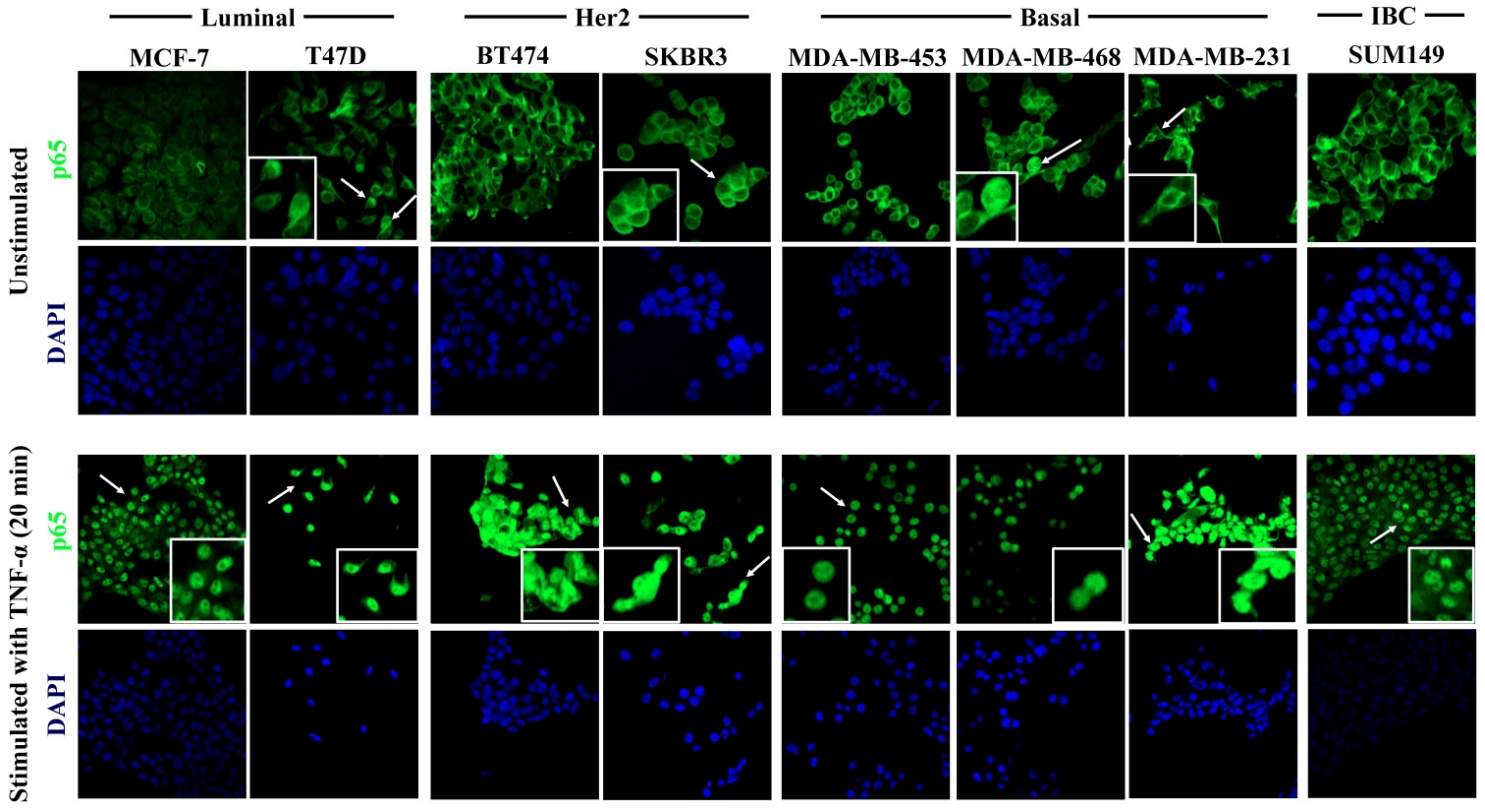
** TNF-α-induced activation of the NF-κB pathway in breast cancer cell lines.** Cells were cultured under control conditions (upper panel) or after 20 min. stimulation with 40 ng/ml TNF-α (lower panel), followed by immunostaining for p65 (green fluorescence) and DAPI nuclear staining (blue). The upper panel shows the analysis of the constitutive cellular localization of p65 in all cell lines. Under control conditions, p65 largely localizes to the cytoplasm. Inserts show magnified areas of rare individual cells characterized by nuclear localization marked with white arrows in the overview panels. The lower panel shows cells after TNF-α stimulation, which induced the nuclear translocation of p65 in all cells studied. Representative images of 20 min of incubation with TNF-α are shown. Original magnification 400X.

In the current study, we report that the expression of a majority of genes related to the NF-κB pathway is associated with better RFS in breast cancer patients. However, we do not know if the expression of these genes largely stems from the tumor cells or from the cells of their microenvironment (e.g. cancer-associated fibroblasts, endothelial cells, pericytes, the immune cell infiltrate, etc.). Therefore, we assessed the relative expression of *IKBKB*, *p50*, *p65*, *NIK*, *RELB*, *IL-8*, and *MMP-1* using quantitative real-time PCR (qPCR) in seven breast cancer cell lines classified according to their molecular subtype: *Luminal*, MCF-7 and T47D; *Her2*, BT474 and SKBR3, and *Basal*, MDA-MB-453, MDA-MB-468 and MDA-MB-231 53, and the inflammatory breast cancer (IBC) cell line SUM149. The qPCR analysis showed two noteworthy results: 1) even when the cell lines were classified in the same molecular subtype, the relative expression (represented by 2^-ΔCt^) of the genes varied among them (consistent with previous data 22) and 2) in the luminal cell line T47D, almost all genes were highly expressed and significantly different from the other cell lines (**Figure 4**). Regardless of T47D, we observed that *RELB* and *IL-8* are more highly expressed in the *basal* and in the IBC line compared with the *Luminal* and *Her2* cell lines. IL-8 showed a particularly high degree of overexpression in the IBC cell line. *p50* and *NIK* were highly expressed in the *basal* cell line MDA-MB-231 and in the IBC cell line SUM-149. Interestingly, MMP-1 was expressed only in the basal cell line MDA-MB-231. We did not find a significant difference in the relative expression of *IKBKB* and *p65* in all cell lines (**Figure 4**). These results suggest that the expression of alternative NF-κB pathway-related genes is more strongly associated with basal subtypes which have a poor outcome 3. To determine if the NF-κB pathway can be activated in the cell lines, we tested whether the p65 subunit of NFκB was present in the cytoplasm (inactive) or nucleus (active) in all breast cancer cell lines in which PCR was performed, by confocal immunofluorescence microscopy. We found that p65 had a cytoplasmic localization in all cell lines, which suggests that the pathway is inactive under our routine cell culture conditions. However, in the T47D, SKBR3, MDA-MB-468, and MDA-MB-231 cell lines, a few cells showed nuclear localization of p65 (**Figure 5, upper panel, white inserts**). To simulate the activation of the pathway by cells of the tumor microenvironment, we treated all cell lines with 40 ng/mL of TNF-α (a known factor for activating the NFκB pathway 54) for 40 min. TNF-α stimulation resulting in a nuclear p65 translocation in all cell lines tested, indicating that the pathway was activated (**Figure 5, lower panel**). We observed nuclear p65 translocation already at 10 min TNF-α treatment (**Supplementary Figure S2**) which persisted for the maximal treatment duration of 40 min (data not shown). These data suggest that some of the cells have a basal low activation of NF-κB that could be further activated in the tumor microenvironment.

### Functional enrichment analysis for NF-κB related proteins

**Figure 6:**
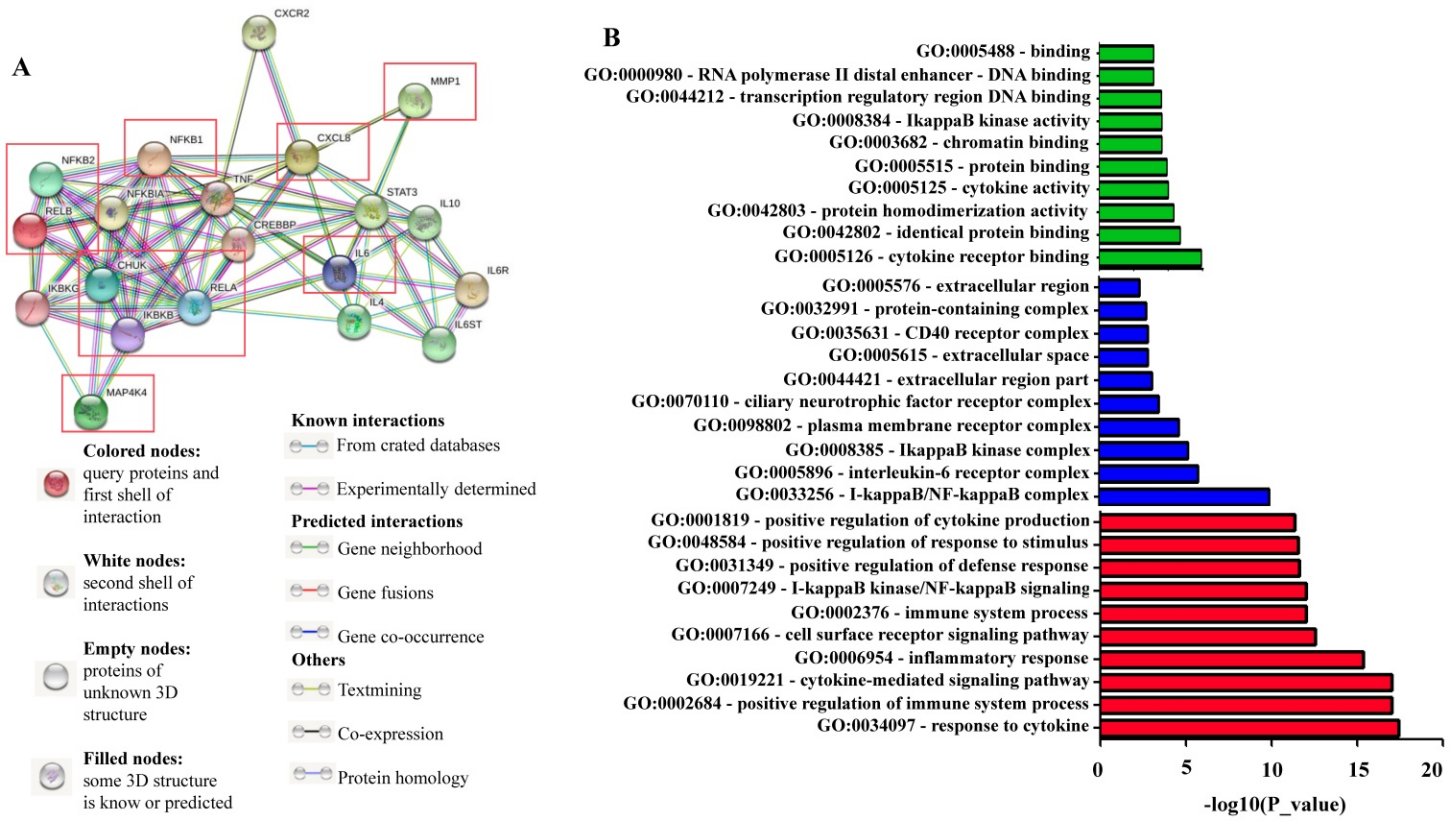

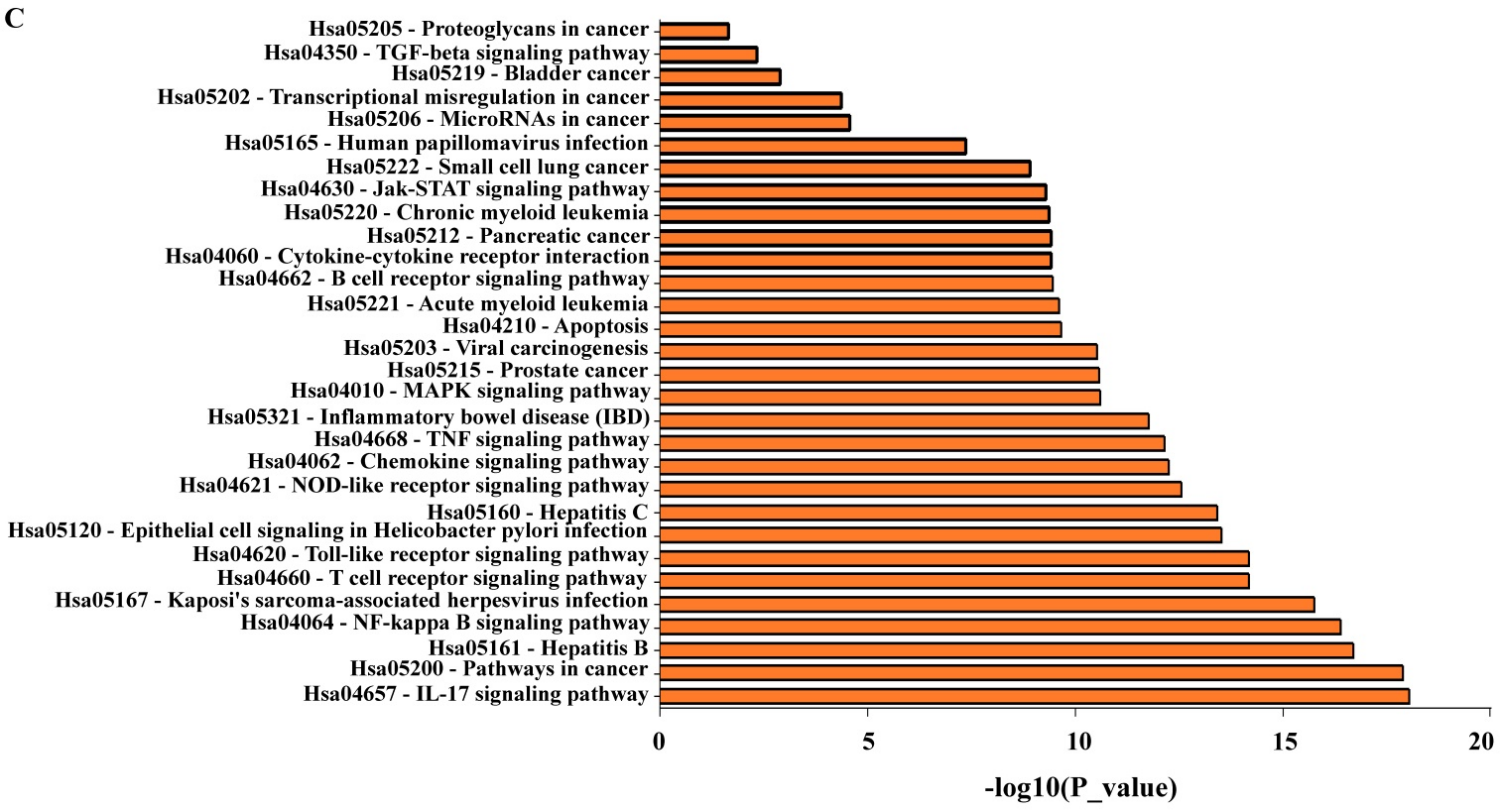
Network of NF-κB protein interactors. A) STRING database output depicting functional and physical interactors of the NF-κB related proteins, IKKα (CHUK), IKBKB, p50/NFKB1, p65/RELA, NIK (MAP4K4), p52 (NFKB2), RELB, IL-8 (CXCL8), IL6, and MMP-1 obtained from http://string-db.org/. The ten NF-κB related proteins are highlighted in red boxes. B) GO (gene ontology) analysis of the NF-κB related proteins. The 10 most significantly (p<0.05) enriched GO terms in molecular function (green), cellular component (blue), and biological process (red) branches are presented. C) KEGG pathway analysis. All the adjusted statistically significant values of the terms were negative 10-base log-transformed.

We used the STRING bioinformatic tool to find biological processes associated with the gene products that we analyzed for their prognostic impact on breast cancer, namely IKKα (CHUK), IKBKB, p50/NFKB1, p65/RELA, NIK (MAP4K4), p52 (NFKB2), RELB, IL-8 (CXCL8), IL6, and MMP-1. The STRING analysis showed that a) all analyzed proteins are highly interconnected, b) the proteins of the NF-κB pathway (IKKα (CHUK), IKBKB, p50/NFKB1, p65/RELA, NIK (MAP4K4), p52 (NFKB2), and RELB) have close interactions with TNF and cAMP-response element-binding protein (CREBBP), and regulate IL-8 (CXCL8), IL-6 and MMP-1. c) NF-κB regulates its own inhibitors of the IκB family such as IKKG (IKKγ) and NFKB1A (IκBα), d) the NF-κB transcriptional target IL-8 (CXCL8) is associated with CXCR2 (IL-8 receptor) and MMP-1, while IL-6 is associated with IL-6 receptor (IL-6R), IL-6 signal transducer (IL6ST), STAT3 and the interleukins IL-4 and IL-10 (**Figure 6A**). We then used GO enrichment analysis to assess molecular function, cellular component and biological process associated with the analyzed proteins. The 10 most significantly enriched terms (p<0.05) in each category are presented in **Figure 6B and Table S5**. Interestingly, KEGG pathway analysis revealed enriched pathways linked to inflammation, cancer, cancer infection origin, miRNAs in cancer and proteoglycans in cancer (**Figure 6C and Table S6**). In addition, STRING developed a statistical co-citation analysis across a large number of scientific texts in PubMed 43. **Table S7** shows the 10 most significantly enrichments, almost all of which are associated with cancer.

Taking into account all these data, our analysis suggests that the expression of specific members of the canonical and alternative NF-κB pathway (*IKKβ, p50/NFKB1, p65/RELA, NIK, p52, RELB, and IL-6*) are associated with a better prognosis in breast cancer patients regardless of their classification (molecular, grade or LN status). Moreover, *IKKα*, *IL-8,* and *MMP-1* expression are associated with poor survival. These genes may represent potential therapeutic targets, especially *IKKα* in ER-positive tumors and IL-8 in the immunomodulatory subtype of TNBCs.

## Discussion

In the present study, the expression of key members of both the canonical and alternative NF-κB pathway and selected downstream targets were analyzed to establish if there was a link between their expression at the transcriptional level and RFS and OS in a large cohort of breast cancer patients. To this purpose, we used the online “Kaplan-Meier plotter” database that allows a real-time analysis to evaluate the prognostic value of those genes in breast cancer. The authors of this tool have combined raw data from several studies to create a single dataset observing that the statistical power was highly increased 27. Before their study in breast cancer, no tool like this was publically available to estimate the prognostic value of a specific gene in a large cohort of clinical patients. Now, this tool has a large number of patients with ovarian, lung, liver and gastric cancer with relapse-free and overall survival data and also miRNA expression in breast and liver cancer (http://kmplot.com/analysis/index.php?p=background) 27. We think that this is a powerful tool to study specific genes relating them to the prognostic of cancer patients.

Signaling pathways are one of the most important players in the normal development of the cell. They control cell growth and differentiation, a process that is inevitably altered in cancer. There are signaling pathways associated with specific processes such as the TGF-β (transforming growth factor-β) pathway associated with the epithelial to mesenchymal transition, a process linked to malignancy 55. However, signaling pathways form extensive networks interconnected with each other that allow important functions in the normal and cancerous cells 56. For example, the induction of late-stage tumor progression is dependent on the cooperation between Ras- and TGF-β-signaling pathways and this also depends critically on NF-κB activity 57. Chronic infection and inflammation are highly oncogenic, and the NF-κB signaling pathway is linked to both processes 17.

In this study, we found that the expression of *IKKα* (associated with both the canonical and alternative NF-κB pathway) and the NF-κB transcriptional targets *IL-8* and *MMP-1* are associated with poor RFS. *IKKα* expression was associated mainly with ER-positive tumors, whereas *IKBKβ*, *p50,* and *p65* from the canonical, and *NIK*, *p52* and *RELB* from the alternative NF-κB pathway were associated with better RFS of breast cancer patients. In agreement with our results, Bennett *et al.* found in immunohistochemical (IHC) analysis of a tissue microarray of 3626 patients with invasive ductal carcinoma of the breast that high cytoplasmic *IKKα* was associated with shorter disease-free survival (also named relapse-free survival) and recurrence-free survival of patients treated with tamoxifen 58. When they analyzed the patients according to the molecular subtype Luminal A, they observed a shorter RFS when *IKKα* was highly expressed in the cytoplasm and the nucleus of tumor cells, and that nuclear expression was associated with ER-positive tumors 58. In another study, Bennet *et al.* also demonstrated that phosphorylated-p65 was associated with worse cancer-specific survival and increased tumor grade 25. Different authors have pointed out that the NF-κB pathway is associated with a poor outcome in another type of cancers: NF-κB/p65 expression was evaluated by IHC in biopsies of 50 patients with rectal cancer undergoing neoadjuvant chemo or radiotherapy and surgery. The results suggested that the high level of NF-κB/p65 is associated with shortened OS 59. In a different study, it was reported that p50^-/-^ mice had smaller tumors and displayed high levels of IL-12 and CXCL10. Importantly, the administration of those cytokines in mice restrained colitis-associated cancer. Interestingly, the authors observed a high density of NK, NKT and CD8^+^ T cells 60. Cai *et al* observed poor survival associated with p50 nuclear expression in diffuse large B-cell lymphoma.

On the other hand, we observed that expression of *IKBKβ*, *p50,* and *p65* from the canonical, and *NIK*, *p52* and *RELB* from the alternative NF-κB pathway were associated with better RFS of breast cancer patients. We were surprised with this finding to set the least and we presently do not have an explanation for this association. It is possible that mRNA expression does not correlate with protein levels or function and that what we are observing is a negative control loop in which high activity leads to less expression. It is also possible that some components of the NF-κB pathway have a stronger effect on promoting tumor growth than others, and as cancer progresses, these less pathogenic components are selected against. In agreement with the former explanation, Bennet et al observed that in spite of lower expression of IKKβ, the knockdown of this protein in breast cancer cell lines increased apoptosis and reduced cell viability 25. On the contrary, in patients with diffuse large B-cell lymphoma of the germinal center subtype and with TP53 mutations, it was found that p50 nuclear expression correlated with significantly better clinical outcome 61. This study agrees with a potentially better prognosis of some of the NF-κB components and with our observation that the expression of *p50* correlated with better RFS in patients with ER-positive and negative breast cancer, Luminal A and even in basal tumors. Differences in the prognostic value may also be a reflection of the dual role of tumor-promoting and anti-tumor effects of inflammation 46.

Other studies support that the alternative pathway of NF-κB is associated with poor prognosis in solid tumors such as glioblastoma, and in mouse models, it has been observed that NF-κB up-regulation is associated with a more aggressive subtype of this disease 62, 63. Also, in prostate cancer, the nuclear expression of RELB was associated with advanced tumors 64, 65. In breast cancer, the patron of NIK expression was analyzed by IHC in the tumor and adjacent non-cancerous tissue specimens of 82 patients. They observed higher expression in cancerous than in adjacent tissues and a significant correlation with lymph node metastasis and lower five-year survival 66. Also, patients with higher expression of *RELB* had decreased RFS and OS in ER-positive breast carcinoma 67. Moreover, in other tumors such as lung cancer, the expression of *NIK* and *RELB* was associated with metastasis and shorter OS 68, 69. These studies are contrary to what we have observed regarding *NIK* and *RELB* mRNA expression.

The measurement of the expression of transcriptional targets of a given signaling pathway may provide a more intuitively accessible assessment of prognostic values. Several proinflammatory cytokines, growth factors, and proteases are regulated by both the classical and alternative NF-κB pathway 16, 17. It is worth noticing that the NF-κB targets analyzed in this study were associated with a poor prognosis suggesting increased activity of the pathway 46, 70-72. We observed an association of IL-8 and MMP-1 expression with poor RFS and OS in breast cancer patients, regardless of ER or nodal status or molecular classification. In the plasma and serum of breast cancer patients, the concentration of IL-8 correlated with poor recurrence-free survival in patients with Her2- tumors and patients with metastasis 73, 74. Using a public database, McGowan and Duffy analyzed the expression of 17 MMPs in the outcome of breast cancer patients. In their study, only MMP-1 was highly expressed in tumors ˃2 cm in size and MMP-1 and other MMPs were associated with poor OS 75. Wang et* al.* demonstrated that MMP-1 expression was associated with poor OS in grade II, nodal-negative, ER-positive and Her2-negative breast cancer using the same database as us 76.

Even though in our study the expression of IL-6 was associated with better survival, its overexpression is associated with tumor growth, progression and response to therapy in many types of cancer 77-80. It has been observed that in patients high serum levels of IL-6 are associated with poor prognosis and shorter survival 81-83. STRING analyses showed that IL-6 is connected with STAT3. The activation of the IL-6/STAT-3 signaling axis apparently is an important event in cancer because it promotes carcinogenesis by regulating multiple survival signaling pathways in tumor cells 84. STAT3 is also an important regulator of immune cell function, it has been linked to cancer 85. Some studies support an NFκB and STAT3 crosstalk required for communication between tumor cells and their microenvironment (reviewed in 86). Our STRING analysis also shows that IL-4, IL-10 are regulated by NFκB. Like IL-6, it has been shown that the expression of IL-4 and IL-10 is associated with cancer 87-89. However, it seems that their greatest participation is not in the tumor cell itself but in regulating its microenvironment. IL-4 and IL-10 together with IL13, M-CSF, and CCL2 participate in the polarization of monocytes to macrophages, whose function is to favor activities such as tissue repair 90. These macrophages in the tumor microenvironment and their chemical mediators favor the processes of invasion, angiogenesis, lymphangiogenesis, intravasation, extravasation, and metastasis 91-93. Among these mediators are CSF-1 (Colony Stimulating Factor-1), GM-CSF (Granulocyte-macrophage- Colony Stimulating Factor), MSP (Macrophage Stimulating Factor), TGF-β, chemokines (CCL2, 3, 4, 7 and 8), MMPs, cathepsins (B and D), VEGF (Vascular Endothelial Growth Factor), and angiopoietin 1 and 2 91-93. These studies suggest that the activation of the NF-κB pathway not only influences the tumor cells but also regulates their microenvironment.

In our study, we do not know to which extent the expression of the genes related to the NFκB pathway stems from the tumor cells or is influenced by the presence of cells in the microenvironment of the tumor biopsies. Therefore, we analyzed the expression of the gene panel mentioned above in different breast cancer cell lines representing the luminal, basal and Her2-positive subtypes. Interestingly, we found that the expression of those genes was different in each cell line, even when they are classified in the same molecular subgroup (**Figure 4**). This could be explained by the huge heterogeneity within the cell lines considering the influence of 1) cancer stem cells (CSCs) 94, 95, 2) clonal evolution 96, and 3) tumor cell plasticity 97, 98. Even with these results, we could observe that mainly genes associated with the alternative NF-κB pathway were prominently expressed in cell lines with more aggressive characteristics. *NIK* and *RELB* were mainly expressed by the basal breast cancer cell line MDA-MB-231. Consistent with these data, the expression of *NIK* has been associated with the proliferation of basal-like subtype of breast cancer 49, 99. Vazquez-Santillan *et al.* described that MDA-MB-231 CSCs showed activation of NF-κB through* NIK* and this was essential for the maintenance of the stemness phenotype 48. However, in acute myelogenous leukemia (AML), it was reported that NIK stabilization acts as a tumor suppressor, and this stabilization induced the alternative pathway of NF-κB. Interestingly, they observed that the expression of *RELA* (p65) was reduced favoring tumor shrinkage. While *RELA* overexpression promoted tumor growth and vice-versa, *RELB* overexpression decreased tumor growth 100. Bennett *et al.* demonstrated that silencing the expression of *IKKα* only affected the proliferation of the Luminal A breast cancer cell line MCF-7 and not the Basal cell line MDA-MB-231 58. In agreement, Park *et al.* demonstrated that *IKKα* participates in the regulation of estrogen-genes such as *cyclin D1* and *c-myc* 101.

Under basal cell culture conditions, the p65 subunit was largely present in the cytoplasm of all cell lines, and only very few cells of aggressive basal cell lines MDA-MB-231 and -468 showed nuclear localization of p65, which would explain why IL-8 and MMP-1 are more highly expressed in the basal cell lines. In agreement with that, Espinoza-Sánchez *et al.* showed that p65 is cytoplasmic in the non-aggressive cell lines MCF-7 and T47D while it is mostly nuclear in the aggressive basal subtype cell lines HS578T and MDA-MB-231 22. Notably, these cells secrete more IL-8 than non-aggressive cells in which p65 showed a cytoplasmic localization 22. They also observed that, after the treatment of MCF-7 and T47D cells with the conditioned media of basal cells, p65 was translocated to the nucleus, emphasizing a role for the tumor microenvironment and of the lateral transmission of malignant properties for NF-κB activation. These induced-aggressive cells increased the expression of chemokine receptors, CSC markers, invasiveness, and exhibited a signaling signature that involved NF-κB activation and the participation of IL-8, MCP-1, G-CSF, GM-CSF, MMP-1, and -2 22, 102. In our study, we observed that after the stimulation with TNF-α all cells translocated p65 to the nucleus, which suggests that NF-κB was activated. Indeed, a previous study demonstrated that after the treatment with TNF-α around 90% of breast cancer cells lines translocated p65 to the nucleus and that this activation correlated with the induction of migration of non-migratory cells 103. Conversely, blocking NF-κB activation inhibited the invasion capacity of the basal cell line MDA-MB-231 103. Interestingly, immunohistochemical detection of nuclear p65 in tumor samples of breast cancer patients was associated with reduced disease-free survival (DFS or RFS) in the same study 103.

## Conclusion

In summary, our results suggest that the expression of NF-κB related genes influences the prognosis of breast cancer patients. Particularly *IKKα*, IL-8, and MMP-1 emerge as important targets for the treatment of BC patients, especially *IKKα* for ER-positive tumors Selective novel compounds targeting the NF-κB pathway may offer a promising therapeutic approach. For example, the FDA (Food and Drug Administration) and the European Medicines Agency approved the proteasome inhibitor Bortezomib for the treatment of multiple myeloma, which also affects the NF-κB pathway 104. Tools like KMplot hold a lot of promise for the study of genes in specific pathways and relating them to patient' survival, pointing out therapeutic targets for more specific treatments. Our study illustrates the need to design studies of signaling pathways at the gene expression and protein levels to understand more about their link to prognostic biomarkers and cancer classification schemes (TNM, grade or molecular). This is a prerequisite for designing personalized treatments that could have a significant economic impact, as the application of unnecessary chemotherapy and its complications such as toxicity, heart problems, and infections will be avoided.

## Supplementary Material

Supplementary figures and tables.

## Figures and Tables

**Table 1:** Clinico-pathological characteristics of the patients investigated in the present study

Parameter	Cohort	Proportion of patients
Array platform	HGU133A	52.1%
	HGU133A plus 2.0	47.9%
ER status	ER-positive	76.4%
	ER-negative	23.6%
HER2 status	HER2 positive	17.7%
	HER2 negative	82.3%
Lymph node status	Node positive	39.2%
	Node negative	60.8%
Grade	Grade 1	14.8%
	Grade 2	42.3%
	Grade 3	42.8%
Molecular subtype (StGallen)	TNBC	17.1%
	Luminal A	48.6%
	Luminal B	27.7%
	HER2 positive	6.5%
Molecular subtype (Pietenpol)	Basal-like 1	19.2% (within TNBC)
	Basal-like 2	7.8% (within TNBC)
	Immunomodulatory	23.4% (within TNBC)
	Mesenchymal	18.4% (within TNBC)
	Mesenchymal stem-like	9.2% (within TNBC)
	Luminal androgen-receptor	22% (within TNBC)
Age	Mean	53.6 years
	Median	53 years
	Range	24-93 years
Relapse-free survival	Follow-up time (months)	72.8+/-46.6
	The proportion of events (relapse)	32%
Overall survival	Follow-up time (months)	84.8+/-47.8
	The proportion of events (death)	25%

**Table 2:** Correlation between members of the NF-κB and survival of breast cancer patients.

Genes		Relapse-free survival (n=3951)		Overall survival (n=1402)	
		HR 95% CI	*P* value	HR 95% CI	*P* value
	*IKKβ*	0.62 (0.62 - 0.77)	1.9e-11*	0.76 (0.61 - 0.94)	0.011*
**Classical pathway**	*p50/NFKB1*	0.63 (0.57 - 0.71)	3.3e-16*	0.67 (0.54 - 0.83)	0.00025*
	*p65/RELA*	0.83 (0.75 - 0.93)	0.00088*	1 (0.81 - 1.24)	1
**Alternative pathway**	*NIK*	0.61 (0.54 - 0.68)	<1E-16*	0.64 (0.52 - 0.8)	7.1e-05*
	*p52*	0.8 (0.72 - 0.89)	5.7e-05*	0.87 (0.7 - 1.07)	0.19
	*RELB*	0.69 (0.62 - 0.78)	6.9e-11*	0.66 (0.53 - 0.82)	0.00019*
**Both pathways**	*IKKα*	1.2 (1.07 - 1.33)	0.0012*	1.19 (0.96 - 1.48)	0.11
**NF-κB target genes**	*IL-8*	1.29 (1.16 - 1.44)	4.1e-06*	1.52 (1.22 - 1.88)	0.00013*
	*IL-6*	0.81 (0.73 - 9.1)	0.00021*	0.92 (0.75 - 1.15)	0.48
	*MMP-1*	1.78 (1.59 - 1.98)	<1E-16*	1.6 (1.29 - 1.99)	1.6e-05*

Bold typing of P-values indicates a significant association (P<0.05). An asterisk indicates an FDR below 10%.

**Table 3:** Correlation between members of the NF-κB and estrogen receptor (ER) status of breast cancer patients.

Genes		ER status	Relapse-free survival				Overall survival	
			Cases	HR 95% CI	*P* value	Cases	HR 95% CI	*P* value
**Classical pathway**	*IKKβ*	Positive	3082	0.74 (0.65 - 0.85)	5.9e-06*	1044	0.78 (0.6 - 1.01)	0.057
		Negative	869	0.85 (0.69 - 1.05)	0.14	358	0.9 (0.61 - 1.33)	0.61
	*p50/NFKB1*	Positive	3082	0.61 (0.54 - 0.69)	4.7e-14*	1044	0.69 (0.53 - 0.89)	0.0046*
		Negative	869	0.67 (0.54 - 0.82)	0.00016*	358	0.58 (0.38 - 0.87)	**0.0072**
	*p65/RELA*	Positive	3082	0.86 (0.75 - 0.97)	0.017*	1044	1.05 (0.81 - 1.36)	0.69
		Negative	869	0.74 (0.6 - 0.91)	0.0049*	358	0.82 (0.55 - 1.21)	0.32
**Alternative pathway**	*NIK*	Positive	3082	0.63 (0.55 - 0.72)	1.4e-12*	1044	0.82 (0.63 - 1.06)	0.12
		Negative	869	0.56 (0.45 - 0.69)	5.3e-08*	358	0.54 (0.36 - 0.81)	0.0022 *
	*p52*	Positive	3082	0.76 (0.67 - 0.86)	2.5e-05*	1044	0.96 (0.75 - 1.24)	0.77
		Negative	869	0.68 (0.55 - 0.84)	3e-04*	358	0.68 (0.46 - 1.01)	0.053
	*RELB*	Positive	3082	0.71 (0.62 - 0.81)	1.4e-07*	1044	0.76 (0.59 - 0.98)	**0.037**
		Negative	869	0.55 (0.44 - 0.68)	3.8e-08*	358	0.47 (0.32 - 0.71)	0.00026*
**Both pathways**	*IKKα*	Positive	3082	1.2 (1.06 - 1.36)	0.0051*	1044	1.09 (0.84 - 1.41)	0.52
		Negative	869	1.09 (0.89 - 1.35)	0.4	358	1.07 (0.72 - 1.58)	0.74
**NF-κB** **target genes**	*IL-8*	Positive	3082	1.18 (1.04 - 1.34)	0.011*	1044	1.36 (1.05 - 1.76)	**0.018**
		Negative	869	1.29 (1.04 - 1.59)	**0.018**	358	1.28 (0.86 - 1.89)	0.22
	*IL-6*	Positive	3082	0.78 (0.68 - 0.88)	0.00012*	1044	0.96 (0.74 - 1.25)	0.76
		Negative	869	0.77 (0.63 - 0.96)	**0.018**	358	1.18 (0.8 - 1.75)	0.4
	*MMP-1*	Positive	3082	1.7 (1.49 - 1.93)	5.6e-16*	1044	1.61 (1.24 - 2.09)	0.00027*
		Negative	869	1.04 (0.84 - 1.28)	0.71	358	1.1 (0.75 - 1.63)	0.62

Bold typing of P-values indicates a significant association (P<0.05). An asterisk indicates an FDR below 10%.

**Table 4:** Correlation between members of the NF-κB and lymph node (LN) status of breast cancer patients.

Genes		LN status		Relapse-Free survival		Overall survival		
			Cases	HR 95% CI	*P* value	Cases	HR 95% CI	*P* value
**Classical pathway**	*IKKβ*	Positive	1133	0.63 (0.52 - 0.77)	5.5e-06*	313	0.65 (0.44 - 0.96)	**0.031**
		Negative	2020	0.81 (0.69 - 0.96)	**0.017**	594	0.71 (0.48 - 1.03)	0.066
	*p50/NFKB1*	Positive	1133	0.75 (0.62 - 0.91)	0.004*	313	0.73 (0.49 - 1.09)	0.12
		Negative	2020	0.73 (0.61 - 0.86)	0.00019*	594	0.62 (0.42 - 0.9)	**0.012**
	*p65/RELA*	Positive	1133	0.91 (0.75 - 1.11)	0.34	313	0.98 (0.67 - 1.45)	0.93
		Negative	2020	1 (0.85 - 1.19)	0.97	594	0.73 (0.5 - 1.08)	0.11
**Alternative pathway**	*NIK*	Positive	1133	0.7 (0.57 - 0.85)	0.00032*	313	0.89 (0.6 - 1.31)	0.55
		Negative	2020	0.78 (0.66 - 0.93)	**0.0048**	594	0.55 (0.37 - 0.82)	0.0026*
	*p52*	Positive	1133	0.67 (0.55 - 0.82)	6.8e-05*	313	0.82 (0.55 - 1.21)	0.32
		Negative	2020	0.83 (0.7 - 0.98)	**0.028**	594	1.16 (0.8 - 1.69)	0.43
	*RELB*	Positive	1133	0.77 (0.63 - 0.94)	0.0092*	313	0.74 (0.5 - 1.09)	0.13
		Negative	2020	0.83 (0.7 - 0.98)	**0.029**	594	0.74 (0.51 - 1.07)	0.11
**Both pathways**	*IKKα*	Positive	1133	0.89 (0.73 - 1.08)	0.24	313	0.74 (0.5 1.09)	0.13
		Negative	2020	1.05 (0.89 - 1.24)	0.59	594	1.12 (0.76 - 1.63)	0.57
**NF-κB** **target genes**	*IL-8*	Positive	1133	1.33 (1.09 - 1.62)	0.0043*	313	1.19 (0.81 - 1.76)	0.37
		Negative	2020	1.35 (1.14 - 1.59)	0.00053*	594	1.18 (0.81 - 1.71)	0.39
	*IL-6*	Positive	1133	1.03 (0.85 - 1.25)	0.78	313	1.07 (0.73 - 1.58)	0.73
		Negative	2020	0.97 (0.82 - 1.15)	0.73	594	0.96 (0.66 - 1.41)	0.84
	*MMP-1*	Positive	1133	1.67 (1.37 - 2.03)	3.5e-07*	313	1.13 (0.76 - 1.66)	0.55
		Negative	2020	1.63 (1.37 - 1.93)	1.8e-08*	594	2 (1.36 - 2.92)	0.00028*

Bold typing of P-values indicates a significant association (P<0.05). An asterisk indicates an FDR below 10%.

## Abbreviations

TME: tumor microenvironment
NF-κB: nuclear factor kappa-light-chain-enhancer of activated B cells
NIK: NF-κB-inducing kinase
RFS: Relapse-free survival
OS: Overall survival
HR: Hazard ratio
MMPs: metalloproteases
IBC: inflammatory breast cancer
ER: estrogen receptor
TNBC: triple-negative breast cancer
LN: lymph node
IHC: immunohistochemistry
qPCR: quantitative real-time PCR
PAMPs: pathogen-associated Molecular Patterns
TLRs: Toll-Like receptors
NLRs: Nod-like receptors
TNF: Tumor Necrosis Factor
RANKL: Receptor Activator of NF-κB ligand
AML: Acute myelogenous leukemia
CSC: cancer stem cells
FDA: Food and Drug Administration
