# Supplementary material for: Differential impact of classical and non-canonical NF-κB pathway-related gene expression on the survival of breast cancer patients
Source: J Cancer. 2019 Aug 28;10(21):5191–211. doi: 10.7150/jca.34302 (PMC6775609; doi:10.7150/jca.34302)
Supplement: Supplementary file 1 — Supplementary figures and tables. [file jcav10p5191s1.pdf]

Supplemental Material

Figure S1

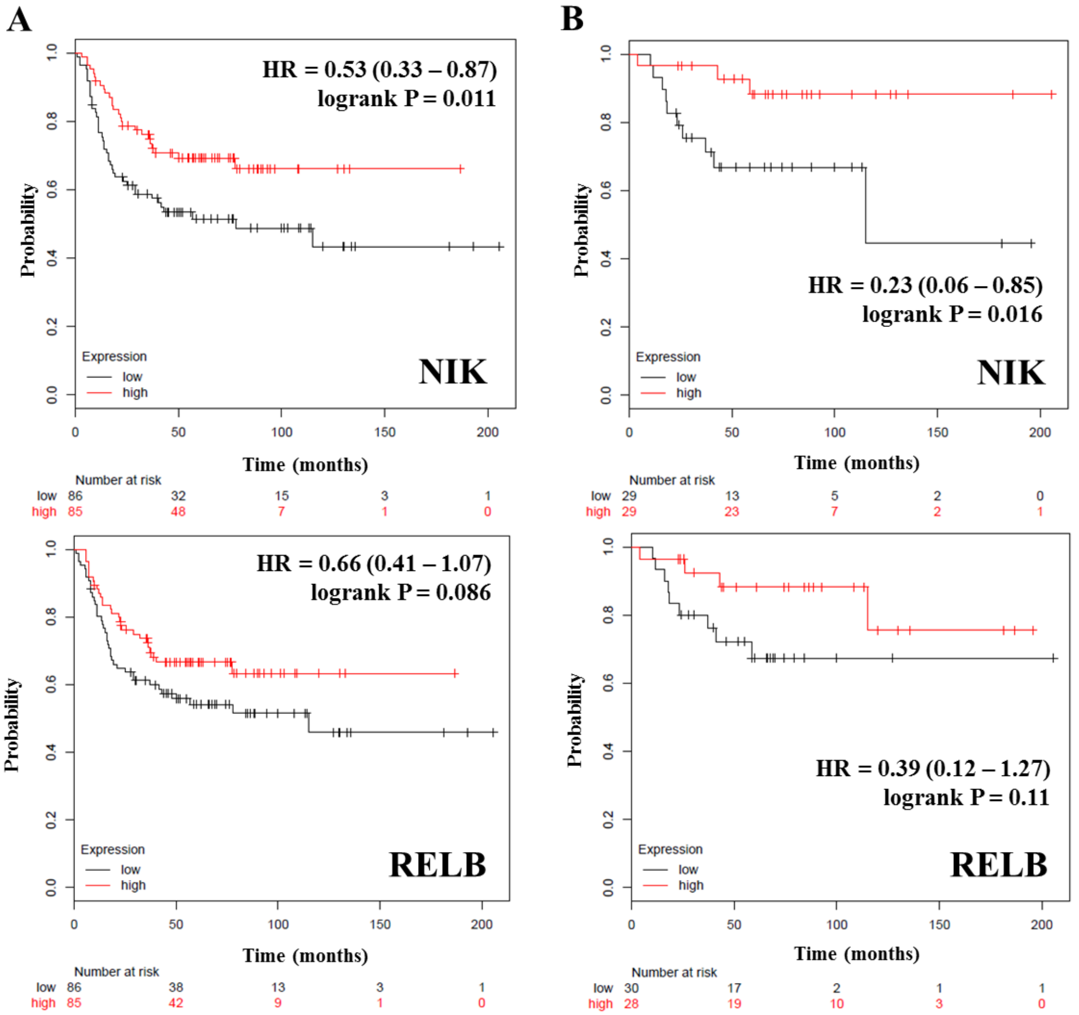

Figure S2

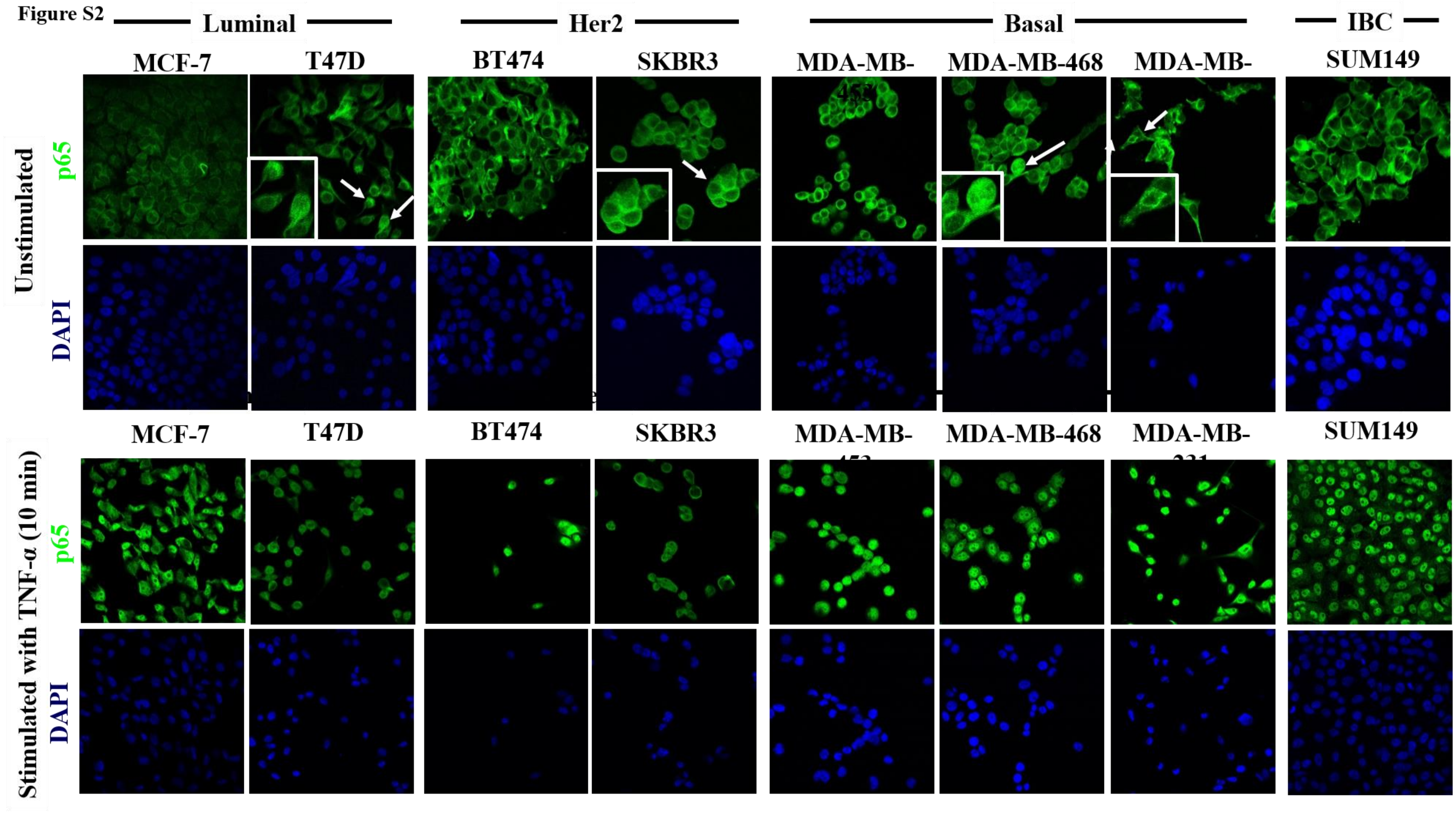
Luminal
Her2
Basal
IBC
MCF-7
T47D
BT474
SKBR3
MDA-MB-
MDA-MB-468
MDA-MB-
SUM149
Unstimulated
p65
DAPI
Stimulated with TNF-α (10 min)

Table S1. Correlation between members of the NF-κB pathway and breast cancer patients classified with the Luminal A molecular subtype.

Genes		Relapse-free survival (n=1933)		Overall survival (n=611)	
		HR 95% CI	*P* value	HR 95% CI	*P* value
**Classical pathway**	*IKKβ*	0.74 (0.62 – 0.88)	**0.00053***	0.97 (0.68 – 1.39)	0.88
	p50/NFKB1	0.58 (0.49 – 0.69)	**8.9e-10***	0.66 (0.46 – 0.95)	**0.024***
	p65/RELA	0.84 (0.71 – 1)	**0.047**	0.95 (0.67 – 1.35)	0.77
**Alternative pathway**	*NIK*	0.6 (0.5 - 0.71)	**6.3e-09***	0.81 (0.57 – 1.16)	0.25
	p52	0.78 (0.66 – 0.93)	**0.0051***	0.9 (0.63 – 1.28)	0.56
	RELB	0.65 (0.54 – 0.77)	**6.2e-07***	0.67 (0.47 – 0.96)	**0.028**
**Both pathways**	*IKKα*	1.2 (1.01 - 1.42)	**0.039**	1.19 (0.83 – 1.71)	0.34
**NF-κB target genes**	*IL-8*	1.2 (1.01 – 1.42)	**0.035**	0.93 (0.65 – 1.33)	0.69
	IL-6	0.77 (0.65 – 0.92)	**0.0031***	0.84 (0.59 – 1.21)	0.35
	MMP-1	1.89 (1.58 – 2.25)	**4.6e-13***	1.72 (1.2 – 2.479	**0.0028***

Bold typing of p-values indicates a significant association (P<0.05). Asterisk indicates a FDR below 10%.

Table S2. Correlation between members of the NF-κB pathway and breast cancer patients classified with the Basal molecular subtype

Genes		Relapse-free survival (n=618)		Overall survival (n=241)	
		HR 95% CI	*P* value	HR 95% CI	*P* value
**Classical pathway**	*IKKβ*	0.89 (0.69 – 1.15)	0.37	0.84 (0.51 – 1.37)	0.47
	p50/NFKB1	0.65 (0.51 – 0.84)	**0.00099***	0.69 (0.42 – 1.14)	0.14
	p65/RELA	0.84 (0.66 – 1.09)	0.19	0.85 (0.52 – 1.4)	0.53
**Alternative pathway**	*NIK*	0.54 (0.42 – 0.7)	**2.4e-06***	0.32 (0.19 – 0.55)	**1.2e-05***
	p52	0.69 (0.53 – 0.89)	**0.0039***	0.71 (0.44 – 1.17)	0.18
	RELB	0.54 (0.42 – 0.7)	**1.8e-06***	0.39 (0.23 – 0.65)	**0.00024***
**Both pathways**	*IKKα*	1.03 (0.8 – 1.33)	0.8	1.07 (0.65 – 1.75)	0.78
**NF-κB target genes**	*IL-8*	1.46 (1.13 – 1.88)	**0.0035***	1.29 (0.79 – 2.12)	0.31
	IL-6	0.83 (0.64 − 1.07)	0.14	1.05 (0.64 − 1.73)	0.84
	MMP-1	1.2 (0.93 – 1.55)	0.15	1.65 (1 – 2.71)	**0.048**

Bold typing of p-values indicates a significant association (P<0.05). Asterisk indicates a FDR below 10%.

Table S3. Correlation between members of the NF-κB pathway and breast cancer patients classified with the Pietenpol immunomodulatory subtype.

Genes		Relapse-free survival (n=203)		Overall survival (n=100)	
		HR 95% CI	*P* value	HR 95% CI	*P* value
**Classical pathway**	*IKKβ*	0.8 (0.44 – 1.44)	0.45	0.72 (0.28 – 1.82)	0.48
	p50/NFKB1	0.51 (0.28 – 0.96)	**0.033**	0.57 (0.21 – 1.52)	0.26
	p65/RELA	0.97 (0.54 – 1.75)	0.92	1.66 (0.64 – 4.3)	0.29
**Alternative pathway**	*NIK*	0.46 (0.25 – 0.86)	**0.013**	0.42 (0.16 – 1.12)	0.074
	p52	0.82 (0.45 – 1.5)	0.53	0.96 (0.38 – 2.41)	0.92
	RELB	1.34 (0.74 – 2.41)	0.33	0.85 (0.34 – 2.17)	0.74
**Both pathways**	*IKKα*	0.83 (0.46 – 1.5)	0.53	0.95 (0.38 – 2.42)	0.92
**NF-κB target genes**	*IL-8*	2 (1.08 – 3.69)	**0.024**	4.11 (1.35- 12.5)	**0.007**
	IL-6	1.13 (0.63 − 2.05)	0.68	1.26 (0.49 − 3.28)	0.63
	MMP-1	0.49 (0.26 – 0.91)	**0.02**	0.48 (0.18 – 1.27)	0.13

Bold typing of p-values indicates a significant association (P<0.05).

Table S4. Correlation between members of the NF-κB pathway and breast cancer patients classified with the grade 3.

**Genes**		**Relapse-free survival (n=903)**		**Overall survival (n=503)**	
		**HR 95% CI**	***P* value**	**HR 95% CI**	***P* value**
**Classical pathway**	*IKKβ*	1.04 (0.84 – 1.3)	0.7	1.09 (0.79 – 1.52)	0.59
	p50/NFKB1	0.75 (0.6 – 0.94)	**0.01***	0.86 (0.62 – 1.2)	0.38
	p65/RELA	0.94 (0.76 – 1.17)	0.59	0.97 (0.7 – 1.35)	0.85
**Alternative pathway**	*NIK*	0.68 (0.55 – 0.85)	**6e-04***	0.6 (0.43 – 0.84)	**0.0024***
	p52	0.75 (0.6 – 0.94)	**0.01***	0.81 (0.58 – 1.13)	0.22
	RELB	0.75 (0.61 – 0.94)	**0.011***	0.71 (0.51 – 0.98)	**0.038**
**Both pathways**	*IKKα*	0.95 (0.77 – 1.19)	0.67	1.06 (0.76 – 1.48)	0.71
**NF-κB target genes**	*IL-8*	1.32 (1.06 – 1.64)	**0.014***	1.36 (0.98 – 1.9)	0.064
	IL-6	0.96 (0.77 – 1.19)	0.7	1.03 (0.74 – 1.44)	0.84
	MMP-1	1.13 (.09 – 1.4)	0.28	1.1 (0.8 – 1.53)	0.56

Bold typing of P-values indicates a significant association (P<0.05). Asterisk indicates a FDR below 10%.

Table S5. GO enrichment analysis associated with the NF-κB related genes. The program STRING was used for this analysis.

Category	Pathway description	Observed gene count	False discovery rate	Matching proteins in your network (labels)
Molecular function	Cytokine receptor binding	7	1.24E-06	CXCL8, IL10, IL4, IL6, IL6ST, STAT3, TNF
	Identical protein binding	11	2.05E-05	CHUK, IKBKB, IKBKG, IL6R, IL6ST, NFKB1, NFKBIA, RELA, RELB, STAT3, TNF
	Protein homodimerization activity	8	4.90E-05	CHUK, IKBKB, IKBKG, IL6R, IL6ST, NFKB1, RELA, STAT3
	Cytokine activity	5	0.0001	CXCL8, IL10, IL4, IL6, TNF
	Protein binding	17	0.00012	CHUK, CREBBP, CXCL8, CXCR2, IKBKB, IKBKG, IL10, IL4, IL6, IL6R, IL6ST, NFKB1, NFKBIA, RELA, RELB, STAT3, TNF
	Chromatin binding	6	0.00024	CREBBP, NFKB1, NFKB2, RELA, RELB, STAT3
	IkappaB kinase activity	2	0.00024	CHUK, IKBKB
	Transcription regulatory region DNA binding	7	0.00025	CREBBP, NFKB1, NFKB2, RELA, RELB, STAT3, TNF
	RNA polymerase II distal enhancer sequence-specific DNA binding	3	0.00072	NFKB1, RELA, RELB
	Binding	20	0.00072	CHUK, CREBBP, CXCL8, CXCR2, IKBKB, IKBKG, IL10, IL4, IL6, IL6R, IL6ST, MAP4K4, MMP1, NFKB1, NFKB2, NFKBIA, RELA, RELB, STAT3, TNF
Cellular component	I-kappaB/NF-kappaB complex	5	6.87E-11	NFKB1, NFKB2, NFKBIA, RELA, RELB
	Interleukin-6 receptor complex	3	1.23E-06	IL6, IL6R, IL6ST
	IkappaB kinase complex	3	4.89E-06	CHUK, IKBKB, IKBKG
	Plasma membrane receptor complex	5	1.78E-05	CHUK, IKBKB, IL6, IL6R, IL6ST
	Ciliary neurotrophic factor receptor complex	2	0.00027	IL6R, IL6ST
	Extracellular region part	8	0.00068	CXCL8, IL10, IL4, IL6, IL6R, IL6ST, MMP1, TNF
	Extracellular space	7	0.0012	CXCL8, IL10, IL4, IL6, IL6R, IL6ST, TNF
	CD40 receptor complex	2	0.0012	CHUK, IKBKB

	Protein-containing complex	12	0.0015	CHUK, CREBBP, IKBKB, IKBKG, IL6, IL6R, IL6ST, NFKB1, NFKB2, NFKBIA, RELA, RELB, STAT3
	Extracellular region	9	0.004	CXCL8, IL10, IL4, IL6, IL6R, IL6ST, MMP1, NFKB1, TNF
**Biological process**	Response to cytokine	18	3.57E-18	CHUK, CXCL8, CXCR2, IKBKB, IKBKG, IL10, IL4, IL6, IL6R, IL6ST, MMP1, NFKB1, NFKB2, NFKBIA, RELA, RELB, STAT3, TNF
	Positive regulation of immune system process	17	1.09E-17	CHUK, CREBBP, CXCL8, CXCR2, IKBKB, IKBKG, IL10, IL4, IL6, IL6R, IL6ST, NFKB1, NFKBIA, RELA, RELB, STAT3, TNF
	Cytokine-mediated signaling pathway	16	1.09E-17	CHUK, CXCL8, CXCR2, IKBKB, IKBKG, IL10, IL4, IL6, IL6R, IL6ST, MMP1, NFKB1, NFKBIA, RELA, STAT3, TNF
	Inflammatory response	14	5.33E-16	CHUK, CXCL8, CXCR2, IKBKB, IKBKG, IL10, IL6, IL6R, NFKB1, NFKB2, RELA, RELB, STAT3, TNF
	Cell surface receptor signaling pathway	18	3.19E-13	CHUK, CREBBP, CXCL8, CXCR2, IKBKB, IKBKG, IL10, IL4, IL6, IL6R, IL6ST, MMP1, NFKB1, NFKBIA, RELA, RELB, STAT3, TNF
	Immune system process	18	1.06E-12	CHUK, CREBBP, CXCL8, CXCR2, IKBKB, IKBKG, IL10, IL4, IL6, IL6R, MMP1, NFKB1, NFKB2, NFKBIA, RELA, RELB, STAT3, TNF
	I-kappaB kinase/NF-kappaB signaling	8	1.06E-12	CHUK, IKBKB, IKBKG, NFKB1, NFKB2, NFKBIA, RELB, TNF
	Positive regulation of defense response	11	2.76E-12	CHUK, CREBBP, IKBKB, IKBKG, IL6, IL6ST, NFKB1, NFKBIA, RELA, RELB, TNF
	Positive regulation of response to stimulus	17	3.13E-12	CHUK, CREBBP, CXCL8, CXCR2, IKBKB, IKBKG, IL4, IL6, IL6R, IL6ST, MAP4K4, NFKB1, NFKBIA, RELA, RELB, STAT3, TNF
	Positive regulation of cytokine production	11	4.66E-12	CHUK, CREBBP, IL10, IL4, IL6, IL6R, IL6ST, NFKB1, NFKB2, RELA, TNF

Table S6. Biological process associated with the NF-κB related genes according to KEEG enrichment analysis. The program STRING was used for this analysis.

Pathway description	Observed gene count	False discovery rate	Matching proteins in your network (labels)
IL-17 signaling pathway	11	8.72E-19	CHUK, CXCL8, IKBKB, IKBKG, IL4, IL6, MMP1, NFKB1, NFKBIA, RELA, TNF
Pathways in cancer	15	1.27E-18	CHUK, CREBBP, CXCL8, IKBKB, IKBKG, IL4, IL6, IL6R, IL6ST, MMP1, NFKB1, NFKB2, NFKBIA, RELA, STAT3
Hepatitis B	11	2.00E-17	CHUK, CREBBP, CXCL8, IKBKB, IKBKG, IL6, NFKB1, NFKBIA, RELA, STAT3, TNF
NF-kappa B signaling pathway	10	4.02E-17	CHUK, CXCL8, IKBKB, IKBKG, NFKB1, NFKB2, NFKBIA, RELA, RELB, TNF
Kaposi's sarcoma-associated herpes-virus infection	11	1.65E-16	CHUK, CREBBP, CXCL8, IKBKB, IKBKG, IL6, IL6ST, NFKB1, NFKBIA, RELA, STAT3
T cell receptor signaling pathway	9	5.89E-15	CHUK, IKBKB, IKBKG, IL10, IL4, NFKB1, NFKBIA, RELA, TNF
Toll-like receptor signaling pathway	9	6.91E-15	CHUK, CXCL8, IKBKB, IKBKG, IL6, NFKB1, NFKBIA, RELA, TNF
Epithelial cell signaling in Helicobacter pylori infection	8	2.89E-14	CHUK, CXCL8, CXCR2, IKBKB, IKBKG, NFKB1, NFKBIA, RELA
Hepatitis C	9	3.60E-14	CHUK, CXCL8, IKBKB, IKBKG, NFKB1, NFKBIA, RELA, STAT3, TNF
NOD-like receptor signaling pathway	9	2.64E-13	CHUK, CXCL8, IKBKB, IKBKG, IL6, NFKB1, NFKBIA, RELA, TNF
Chemokine signaling pathway	9	5.30E-13	CHUK, CXCL8, CXCR2, IKBKB, IKBKG, NFKB1, NFKBIA, RELA, STAT3
TNF signaling pathway	8	7.03E-13	CHUK, IKBKB, IKBKG, IL6, NFKB1, NFKBIA, RELA, TNF
Inflammatory bowel disease (IBD)	7	1.69E-12	IL10, IL4, IL6, NFKB1, RELA, STAT3, TNF

MAPK signaling pathway	9	2.40E-11	CHUK, IKBKB, IKBKG, MAP4K4, NFKB1, NFKB2, RELA, RELB, TNF
Prostate cancer	7	2.60E-11	CHUK, CREBBP, IKBKB, IKBKG, NFKB1, NFKBIA, RELA
Viral carcinogenesis	8	2.86E-11	CREBBP, IKBKG, IL6ST, NFKB1, NFKB2, NFKBIA, RELA, STAT3
Apoptosis	7	2.10E-10	CHUK, IKBKB, IKBKG, NFKB1, NFKBIA, RELA, TNF
Acute myeloid leukemia	6	2.33E-10	CHUK, IKBKB, IKBKG, NFKB1, RELA, STAT3
B cell receptor signaling pathway	6	3.34E-10	CHUK, IKBKB, IKBKG, NFKB1, NFKBIA, RELA
Cytokine-cytokine receptor interaction	8	3.73E-10	CXCL8, CXCR2, IL10, IL4, IL6, IL6R, IL6ST, TNF
Pancreatic cancer	6	3.91E-10	CHUK, IKBKB, IKBKG, NFKB1, RELA, STAT3
Chronic myeloid leukemia	6	4.33E-10	CHUK, IKBKB, IKBKG, NFKB1, NFKBIA, RELA
Jak-STAT signaling pathway	7	5.22E-10	CREBBP, IL10, IL4, IL6, IL6R, IL6ST, STAT3
Small cell lung cancer	6	1.23E-09	CHUK, IKBKB, IKBKG, NFKB1, NFKBIA, RELA
Human papillomavirus infection	7	4.33E-08	CHUK, CREBBP, IKBKB, IKBKG, NFKB1, RELA, TNF
MicroRNAs in cancer	4	2.70E-05	CREBBP, IKBKB, NFKB1, STAT3
Transcriptional misregulation in cancer	4	4.25E-05	CXCL8, IL6, NFKB1, RELA
Bladder cancer	4	0.0013	CXCL8, MMP1
TGF-beta signaling pathway	2	0.0047	CREBBP, TNF
Proteoglycans in cancer	2	0.022	STAT3, TNF

Table S7. Statistical co-citation analysis associated with the NF-κB related genes according to PubMed. The program STRING was used for this analysis.

Pathway description	Observed gene count	False discovery rate	Matching proteins in your network (labels)
(2009) Current and potential inflammation targeted therapies in head and neck cancer.	13	5.89E-25	CHUK, CXCL8, IKBKB, IL10, IL4, IL6, IL6R, NFKB1, NFKB2, RELA, RELB, STAT3, TNF
(2014) NF-KB, an active player in human cancers.	13	3.11E-23	CHUK, CXCL8, IKBKB, IKBKG, IL10, IL6, NFKB1, NFKB2, NFKBIA, RELA, RELB, STAT3, TNF
(2017) STAT3 and NF-KB are Simultaneously Suppressed in Dendritic Cells in Lung Cancer.	13	5.32E-22	CHUK, CXCL8, IKBKB, IKBKG, IL10, IL4, IL6, NFKB1, NFKB2, RELA, RELB, STAT3, TNF
(2011) Cell Autonomous and Non-Autonomous Functions of IKKBeta and NF-KB during the Pathogenesis of Gastrointestinal Tumors.	11	3.48E-21	CHUK, CXCL8, IKBKB, IKBKG, IL6, NFKB1, NFKB2, NFKBIA, RELA, RELB, TNF
(2018) Inhibition of NF-KB prevents the acidic bile-induced oncogenic mRNA phenotype, in human hypopharyngeal cells.	12	4.87E-21	CHUK, IKBKB, IKBKG, IL10, IL6, NFKB1, NFKB2, NFKBIA, RELA, RELB, STAT3, TNF
(2015) The Role of the Transcriptional Regulation of Stromal Cells in Chronic Inflammation.	13	1.5E-20	CHUK, CXCL8, IKBKB, IKBKG, IL6, IL6R, MMP1, NFKB1, NFKB2, RELA, RELB, STAT3, TNF
(2016) IKKNF-KB signaling contributes to glioblastoma stem cell maintenance.	11	4.03E-20	CHUK, CXCL8, IKBKB, IKBKG, IL6, NFKB1, NFKB2, NFKBIA, RELA, RELB, TNF
(2012) A key role for NF-KB transcription factor c-Rel in T-lymphocyte-differentiation and effector functions.	11	1.93E-19	CHUK, IKBKB, IKBKG, IL4, IL6, NFKB1, NFKB2, RELA, RELB, STAT3, TNF
(2015) Transcriptional Activation of Inflammatory Genes: Mechanistic Insight into Selectivity and Diversity.	12	1.94E-19	CREBBP, CXCL8, IKBKG, IL10, IL4, IL6, NFKB1, NFKB2, NFKBIA, RELA, RELB, TNF
(2016) miR-130b, an onco-miRNA in bladder cancer, is directly regulated by NF-KB and sustains NF-KB activation by decreasing Cylindromatosis expression.	10	2.95E-19	CHUK, CXCL8, IKBKB, IKBKG, NFKB1, NFKB2, NFKBIA, RELA, RELB, TNF

Table S8. PCR Primer sequences

Gene	Primer sequences	
	**Forward primer**	**Reverse primer**
IKKβ	TCCGATGGCACAATCAGGAAAC	TCCAGGCACCACCGCTCTC
p65	GACCTGAATGCTGTGCGGC	ATCTTGAGCTCGGCAGTGTT
p50	TCCACAAGGCAGCAAATAGA	GGGGCATTTTGTTGAGAGTT
NIK	TTAGAAACACCCCGCAGTTC	TCCACACGTGGTTCAGACAT
RELB	CACTCTCGCTCGCCGTTTC	CGAAGCCGTTCTCCTTGATGT
IL-8	GTCATTGCCAGCTGTGTTGGT	TGACTGTGGAGTTTTGGCTGTTT
MMP-1	CGACTCTAGAAACACAAGAGCAAGA	AAGGTTAGCTTACTGTCACACGCTT
SNAI-1	CGAGCCCAGGCAGCTATTTC	CCCGACAAGTGACAGCCATT
STAT3	CTTTGAGACCGAGGTGTATCACC	GGTCAGCATGTTGTACCACAGG
β-ACTIN	TCAAGATCATTGCTCCTCCTGAG	ACATCTGCTGGAAGGTGGACA
